# Neighborhood-Scale O_3_, NO_2_, and PM_2.5_ in Washington, D.C. from a 1 × 1 km^2^ WRF–SMOKE–CMAQ Framework

**DOI:** 10.1021/acsestair.6c00155

**Published:** 2026-09-04

**Authors:** Hoda Hallaji, Siqi Ma, Daniel Q. Tong, Lucas R. F. Henneman

**Affiliations:** † Civil, Environmental, and Infrastructure Engineering Department, 3298 George Mason University, Fairfax, Virginia 22030, United States; ‡ Atmospheric, Oceanic & Earth Sciences Department, 3298 George Mason University, Fairfax, Virginia 22030, United States

**Keywords:** CMAQ, high-resolution air quality modeling, urban air pollution, O_3_, NO_2_, PM_2.5_, population-weighted exposure

## Abstract

Quantifying air pollution concentrations at fine spatial scales is important for resolving intra-urban exposure variability and identifying sources that disproportionately impact population groups across locations. We developed a neighborhood-scale resolution (1 × 1 km^2^) WRF–SMOKE–CMAQ modeling framework to characterize O_3_, NO_2_, and PM_2.5_ concentrations in Washington, D.C. in 2017. In evaluations against EPA Air Quality System observations and the National Climatic Data Center meteorological data, WRF reproduced the temporal variability of temperature and specific humidity with reasonable agreement (average *r* = 0.8 and 0.7, respectively), although biases varied across seasons. CMAQ reproduced seasonal and diurnal patterns of O_3_ and PM_2.5_ with r up to 0.7 for hourly O_3_, though NO_2_ was negatively biased (−14% to −29%), reflecting challenges in resolving near-road gradients. We identified pronounced intra-urban concentration variability of NO_2_, whose coefficient of variation was greater than that of O_3_ and more than of three times that of PM_2.5_. O_3_ concentrations were lower in all months by 4% to 11% in the urban core compared to outside the urban core due to NO_*x*_ titration/inhibition. Relative to 4 × 4 km^2^ resolution, 1 × 1 km^2^ simulations increased population-weighted exposure (PWE) for O_3_ (+0.5% to +1.5%) but decreased PWE for NO_2_ (−3% to −6%) and PM_2.5_ (−1.5% to −3%). VOC/NO_*x*_ ratios indicated predominantly NO_*x*_-saturated chemistry within the urban core. This work presents the value and the remaining challenges of high-resolution regional air quality modeling in heterogeneous urban settings.

## Graphical abstract

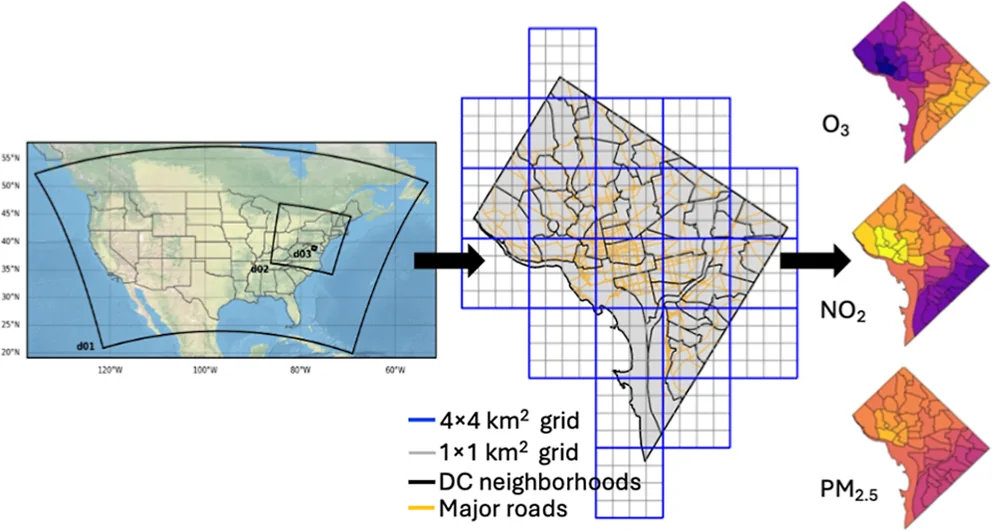

## 1 Introduction

Air pollution is a leading environmental risk factor for human health in the United States and globally. Exposure to ambient ground-level ozone (O_3_), nitrogen dioxide (NO_2_) and fine particulate matter (PM_2.5_) has been associated with multiple health end points, including increased incidence of cardiovascular and respiratory disease, cognitive decline, and premature mortality.^1^ ^,^ ^2^ ^,^ ^3^ For example in the US, about 70,000 deaths were attributable to PM_2.5_ exposure in 2023.^4^ Beyond these aggregate statistics, air pollution contributes to structural inequalities in health outcomes, as communities of color and low-income neighborhoods often experience significantly higher pollutant exposure levels and health burden than their wealthier or white counterparts.^5^ ^,^ ^6^ ^,^ ^7^ These disparities are particularly visible in urban areas, where pollution levels can vary by several-fold over distances of just a few kilometers.^8^

Urban environments such as Washington D.C. are multi-source systems in which emission hotspots, traffic corridors, microclimates, and topography all interact to create localized air pollution patterns. Understanding air pollution spatial and temporal gradients is important for identifying vulnerable populations, evaluating the effectiveness of mitigation strategies, and ensuring equitable access to clean air.^8^ Fine spatial-scale exposure estimates can resolve intra-urban variability and enable a more complete characterization of exposure heterogeneity within cities.^9^ ^,^ ^10^

While established regulatory air quality monitors provide consistent temporal coverage, they are spatially sparse and unevenly distributed, with fewer monitors in low-income and underserved communities.^11^ ^,^ ^12^ A typical U.S. metropolitan area may contain only a handful of monitors, which are generally located to meet compliance requirements rather than to capture spatial heterogeneity. Satellite remote sensing offers relatively fine-scale spatial–temporal coverage but has its own limitations, especially for near-surface pollutants like NO_2_ and PM_2.5_, due to cloud interference, vertical resolution constraints, and the coarse revisit times of polar-orbiting platforms,^13,14^ (geostationary satellites such as TEMPO over North America offer finer temporal coverage). As a result, air pollution exposure differences within urban populations remains under-characterized.

Chemical transport models (CTMs) simulate atmospheric processes including emissions, advection, diffusion, deposition, and chemical transformation to produce spatially complete fields of pollutant concentrations. CTMs can address many of the gaps inherent in other exposure assessment methods.^15^ ^,^ ^16^ These models estimate air quality in locations that are not monitored and can be used to conduct scenario analyses for regulatory or planning purposes. Coarse-resolution simulations (e.g., 12 × 12 km^2^ grids) may perform well for regional-scale transport but average over within-city variation. In contrast, finer-scale simulations (1 × 1 to 4 × 4 km^2^) can capture neighborhood-level gradients in pollutant concentrations but require detailed inputs and more computing power and storage.^16^ ^,^ ^17^

Washington, D.C. is a dense metropolitan area that presents a complex mix of emission sources and meteorological influences. Previous studies have shown large disparities in PM_2.5_-attributable mortality and disease rates across the city, with higher incidence observed in the eastern half of the city than the west.^7^ ^,^ ^8^ Resolving local-scale air quality in D.C. is important to accurately characterize pollution spatial variability and assess exposure inequalities. The Washington, DC–MD–VA metropolitan area is currently designated as a moderate nonattainment area for the 2015 8 h O_3_ National Ambient Air Quality Standards (NAAQS).^18^ This designation reflects ongoing challenges related to both local emissions and regional transport of O_3_ precursors and necessitates coordinated emission control strategies across jurisdictions.^19^ For other criteria pollutants such as PM_2.5_, the region is generally classified as attainment or maintenance under current NAAQS, highlighting O_3_ as the dominant regulatory concern for air quality management in the region.^20^

The D.C.-Baltimore region has been the subject of several intensive ground-based and aircraft measurement campaigns, and researchers have paired the measurements with CTMs and dispersion models to understand drivers of O_3_, NO_2_, and PM_2.5_.^21^ ^,^ ^22^ These studies have shown that air quality in the region is strongly influenced by interactions among local emissions, regional transport, and coastal meteorology.^22^ ^,^ ^23^ In particular, Chesapeake Bay breeze circulations can recirculate O_3_ and its precursors, stagnation events can enhance concentrations of primary and secondary pollutants, and the regions’ local sources contribute to substantial spatial variability in pollutant concentrations over relatively short distances.^24^ O_3_ chemistry over the region has lately been in the NO_*x*_-sensitive regime, suggesting that NO_*x*_ emissions reductions have potential to reduce O_3_ concentrations, but the most urban areas may still be NO_*x*_ saturated and subject to titration/O_3_ inhibition from fresh NO_*x*_.^25^ ^,^ ^23^ Aircraft observations during the DISCOVER-AQ campaign further demonstrated substantial vertical and horizontal variability in trace gases and PM_2.5_ across the Baltimore-Washington metropolitan area, highlighting the importance of resolving neighborhood-scale gradients and boundary-layer processes when evaluating pollutant exposure and atmospheric chemistry.^23^ ^,^ ^26^ CTMs may have the ability to resolve such urban photochemistry dynamics;^27^ however, previous studies in Washington D.C. region have primarily relied on field observations or coarser-resolution modeling. Consequently, neighborhood-scale spatial variability in pollutant concentrations and O_3_ chemical regimes has not been systematically characterized, leaving an important gap in understanding local air quality and exposure patterns.

This study evaluates the extent to which a 1 × 1 km^2^ resolution WRF-SMOKE-CMAQ model system resolves urban-rural concentration differences and neighborhood-scale exposure patterns compared with a 4 × 4 km^2^ resolution domain. To make this comparison and support an exposure assessment in Washington, D.C., we developed and applied a CTM modeling system with a 1 × 1 km^2^ grid resolution. By producing neighborhood-scale exposure estimates, we aim to provide a foundation for supporting air quality management in the region by understanding air pollution spatial–temporal variability and potential strategies to mitigate elevated air pollution.

## 2 Method

We used the Community Multi-scale Air Quality (CMAQ, v5.4,^28^) model with a nested domain setup: a 12 × 12 km^2^ grid covered the contiguous U.S., a 4 × 4 km^2^ grid covered the eastern U.S., and a 1 × 1 km^2^ grid for the D.C. region (Figure 1). Meteorological inputs were developed using the Weather Research and Forecasting (WRF, v4.2.1,^29^) model, and emissions were processed using the Sparse Matrix Operating Kernel of Emissions (SMOKE, v4.7,^30^), based on the 2017 National Emissions Inventory (NEI). The standalone WRF and SMOKE outputs were then used as inputs for the CMAQ model.

**Figure 1:**
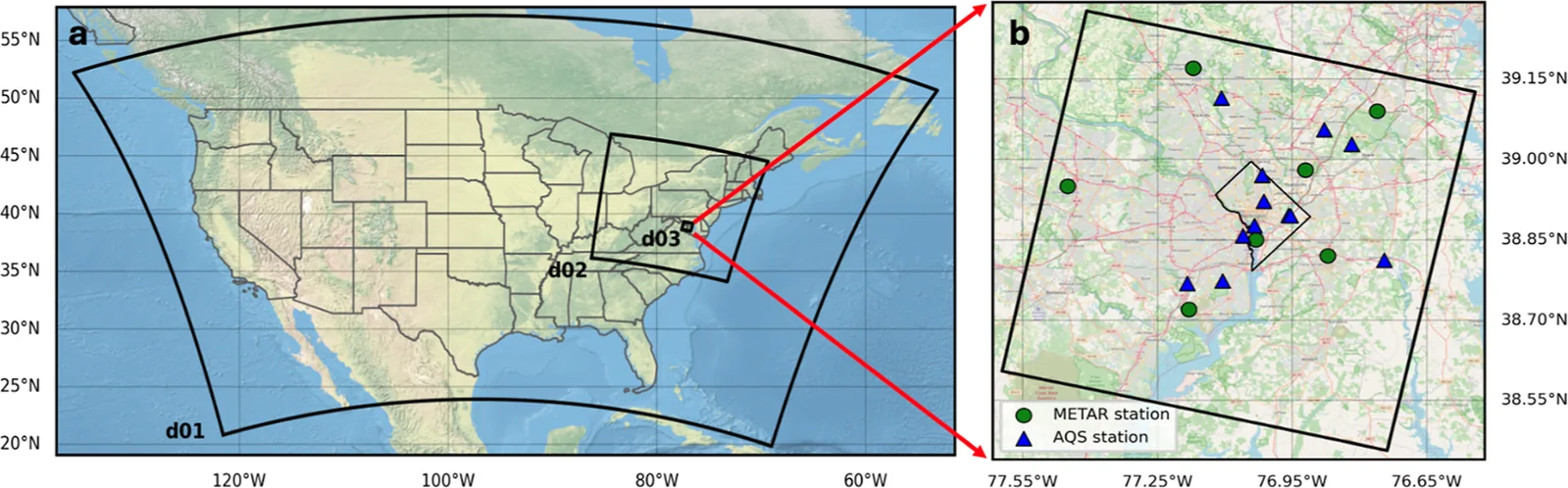
Nested model domains and observation sites. (a) Spatial extent of the three nested model domains (d01, d02, d03), with the innermost 1 × 1 km^2^ domain (d03) centered on Washington, D.C. (b) Enlargement of domain d03 showing METAR weather stations, whose hourly observations are compiled by the National Climatic Data Center (NCDC), together with monitoring sites from the United States Environmental Protection Agency Air Quality System (AQS) used for model evaluation. Road network data © OpenStreetMap contributors.^35^

### 2.1 Meteorology Inputs

WRF simulations covered four months in 2017: February, May, August, and November. A 10 day spin-up period was employed to allow soil moisture and temperature to equilibrate with observational constraints, and soil moisture initialization was activated during this phase.^31^ We used a 60 s timestep for the 12 × 12 km^2^ domain, 20 s for the 4 × 4 km^2^ domain, and 5 s for the 1 × 1 km^2^ domain. The nested domains employed integer parent-grid ratios of 3:1 and 4:1, resulting in a 12 × 12 km^2^ to 4 × 4 km^2^ and then to 1 × 1 km^2^ nesting configuration. The WRF model was configured with 35 vertical layers and a model-top pressure of 50 hPa, with the lowest model level thickness around 20 meters. Initial and 3-hourly lateral boundary conditions for the 12 × 12 km^2^ domain were sourced from the North American Regional Reanalysis (NARR).^32^ We used the Single-Moment 3-Class Simple Ice microphysics scheme,^33^ and the Kain-Fritsch cumulus parameterization (CU_PHYSICS = 1),^34^ the YSU planetary boundary layer scheme, the Revised MM5 (Jimenez) surface layer scheme, and the Thermal Diffusion land surface model. Land-use characteristics were represented using the MODIFIED_IGBP_MODIS land-use classification. The WRF simulations were performed without Four-Dimensional Data Assimilation (FDDA) to avoid negating fine-resolution benefits by nudging to course reanalysis data, and model evolution was governed by the specified initial and lateral boundary conditions. The domain feedback mechanism was enabled, and the model included horizontal and vertical damping to prevent instability. No urban canopy parameterization was employed in the simulations; this is discussed in the Limitations section.

### 2.2 Emissions Inputs

All emission sources were spatially and chemically distributed using surrogates in SMOKE, which additionally assigned chemical profiles. Point-source emissions were obtained from the U.S. Environmental Protection Agency’s 2017 National Emissions Inventory (NEI 2017).^36^ Gridded 1 × 1 km^2^ grid resolution emissions were from the Neighborhood Emission Mapping Operation (NEMO) dataset,^37^ which were derived from NEI 2017 using 108 spatial surrogates representing demographic, transportation, land-use, and industrial activity indicators. The inventory incorporates meteorology-dependent temporal profiles, allowing emissions from sectors such as fugitive dust, residential wood combustion, and agriculture to vary dynamically with atmospheric conditions. Meteorology-dependent emissions were used in the original NEMO framework and were not updated using the WRF simulations in this study. Biogenic emissions, lightning-generated NO_*x*_ emissions, and windblown dust were computed “inline” in CMAQ. The 1 × 1 km^2^ domain directly utilized the native NEMO emissions inventory. Emissions for the 4 × 4 km^2^ domain were generated by spatially aggregating the native 1 × 1 km^2^ NEMO emissions to the 4 × 4 km^2^. Emissions for the 12 × 12 km^2^ domain were obtained from EPA’s EQUATES project.^38^

### 2.3 Air Quality Model

We implemented CMAQ in a one-way nested configuration. For the nested simulations, boundary conditions were generated from the parent-domain CMAQ simulations, the 12 × 12 km^2^ simulation provided boundary conditions for the 4 × 4 km^2^ domain, and the 4 × 4 km^2^ simulation provided boundary conditions for the 1 × 1 km^2^ domain. We used the Carbon Bond 6 Revision 5 gas-phase mechanism coupled with the AERO7 aerosol module (*cb6r5_ae7_aq*), consistent with the 2017 emissions platform.

WRF outputs were preprocessed for CMAQ using the Meteorology–Chemistry Interface Processor (MCIP, v5.4) to generate hourly meteorological inputs required by CMAQ. These inputs included three-dimensional fields such as air temperature, water vapor mixing ratio, pressure, dry air density, layer heights, cloud fraction, cloud water, and rainwater, as well as two-dimensional surface, land-use, soil, and boundary-layer variables. Boundary and initial conditions for the outermost 12 × 12 km^2^ CMAQ domain were derived from EPA’s EQUATES CMAQ modeling database and processed using the CMAQ ICON and BCON preprocessors.^38^ ^,^ ^39^ Each simulation employed the default dynamic time-stepping algorithm in CMAQ, which adjusts the computational timestep based on Courant–Friedrichs–Lewy (CFL) stability criteria to ensure numerical stability across nested domains.

### 2.4 Model Evaluation

We report evaluation results for the 1 × 1 km^2^ domain, comparing modeled WRF meteorology and CMAQ concentrations with available ground observations. Model–observation agreement is summarized with standard statistics (Table S1): normalized mean bias (NMB), correlation coefficient (r), mean error (ME), mean bias (MB), and root-mean-square error (RMSE). Model performance is assessed using the benchmark criteria and goal levels provided by Emery et al. (2017).^40^

We evaluated WRF temperature and specific humidity at 2 m (T2 and Q2) and wind speed (WS) and wind direction (WD) at 10 m against hourly Meteorological Aerodrome Report (METAR) measurements compiled by the National Climatic Data Center (NCDC). Q2 is used instead of relative humidity because it is a mass-based humidity variable that responds more smoothly to model temperature errors and therefore provides a more robust indicator of moisture performance.^41^ Comparisons are carried out for the 7 WRF grid cells that contain NCDC stations (Figure 1b), providing observational comparisons for approximately 0.1% of the 5929 (77*77) simulation grid cells.

Model performance for O_3_, NO_2_ and PM_2.5_ in the 1 × 1 km^2^ CMAQ domain was evaluated following the recommended operational–diagnostic–dynamic framework^42^ using hourly observations from EPA’s Air Quality System (AQS) (Figures 1b and S1). Daily metrics (average NO_2_, maximum daily 8 h average O_3_, average O_3_, and average PM_2.5_) were constructed from hourly metrics. Within the domain, the number of active monitors varied by pollutant and season: O_3_ was measured at 5 sites in February and 8 sites in the remaining months, NO_2_ was measured at 7 sites in each month, and PM_2.5_ was measured at 6–8 sites depending on the month (Table S2 and Figure S1). Prior to analysis, hourly AQS observation data marked with quality-assurance flags were removed.

### 2.5 Population-Weighted Exposure

Population counts at the census block group were obtained from the American Community Survey for 2017 ^43^ and spatially allocated to neighborhood clusters based on the proportion of block group area within each cluster (Figure S2). D.C. uses the neighborhood clusters for community planning and other purposes. In addition to their political relevance, neighborhood clusters balance spatial detail with statistical stability and policy relevance. While census block groups provide finer resolution than neighborhood clusters, their small size and variability relative to the 1 × 1 km CMAQ grid can introduce artificial spatial variability; area-weighting to clusters reduces this mismatch and yields interpretable exposure estimates.^44^ CMAQ concentrations of daily maximum 8 h average (MDA8) O_3_ and daily mean O_3_, NO_2_ and PM_2.5_ were aggregated to a neighborhood cluster using area-weighted averaging of intersecting CMAQ grid cells.

We calculated population-weighted exposure (PWE,^45,46^ ) using CMAQ ambient air pollution concentrations and Washington, D.C. neighborhood cluster populations. For a given pollutant *p*, PWE is defined as

1 $${PWE}_{p}=\frac{\sum_{i}C_{i,p}P_{i}}{\sum_{i}P_{i}}$$

where *C*_*i*,*p*_ is the mean concentration of pollutant *p* within neighborhood cluster *i*, and *P*_*i*_ is the total residential population of that cluster. This approach yields a single daily exposure value for each pollutant.

## 3 Results

### 3.1 WRF Model Evaluation

WRF performance is generally consistent with previous applications for air quality modeling, with varying performance among meteorological variables and evaluation metrics (Table 1). While some evaluation metrics fall outside the criteria defined by Emery Tai (2001),^41^ the model generally reproduces the observed seasonal and meteorological patterns, with better performance for temperature and mixing ratio than for wind-related variables. For near-surface temperature (T2), correlations are relatively high across all months (*r* = 0.7–0.9), indicating that the model generally reproduces the observed temporal variability. However, because temperature exhibits a strong diurnal cycle that is largely driven by solar forcing, correlation alone does not necessarily indicate high model skill and should be interpreted together with bias and error statistics. Domain-wide mean errors of 2.9 K and RMSEs of 3.7 K indicate substantial deviations at individual locations and times, and are somewhat larger than a similar-resolution application of WRF-CMAQ in Chicago.^27^ These elevated RMSE values may reflect neighborhood-scale meteorological variability that is not parameterized in our WRF configuration. A cold bias is observed in February (MB = −2.5 K), while warm biases are found in August (MB = +1.1 K) and November (MB = +1.9 K). In May, T2 bias is small (MB = −0.2 K), although ME is slightly above the recommended benchmark.

**Table 1:** Hourly WRF Meteorological Performance for February, May, August, and November 2017 Over the 1 × 1 km^2^ Domain Assessed at 7 NCDC Monitors^a^

Var	month	N (hourly paired)	MB	ME	RMSE	*r*
T2 (K)	02/2017	4533	–2.5*	3.2*	4.0	0.9
	05/2017	4280	–0.2	3.4*	4.4	0.7
	08/2017	4985	1.1*	2.0	2.7	0.8
	11/2017	4774	1.9*	2.9*	3.7	0.9
	average	4643	0.1	2.9	3.7	0.8
	criteria	-	±0.5	±2	n/a	n/a
Q2(g/kg)	02/2017	4532	0.4	0.8	1.1	0.9
	05/2017	4278	–0.7	2.1*	2.9	0.6
	08/2017	4962	–1.6*	2.9*	3.5	0.5
	11/2017	4754	0.7	1.2	1.4	0.9
	average	4632	–0.3	1.8	2.2	0.7
	criteria	-	±1	±2	n/a	n/a
WS(m/s)	02/2017	4533	–0.5	1.6	2	0.6
	05/2017	4282	–0.3	1.7	2.3*	0.3
	08/2017	5002	–0.3	1.4	1.8	0.2
	11/2017	4774	–0.3	1.5	1.9	0.4
	average	4648	–0.3	1.5	1.9	0.4
	criteria	-	±0.5	n/a	2	n/a
WD (°)	02/2017	3391	4.9	28.4	43.7	0.7
	05/2017	3255	10.6*	67.6*	84.1	0.3
	08/2017	2851	7.9	67.2*	84.1	0.3
	11/2017	3169	9.4	30.6*	44.9	0.5
	average	3167	8.2	48.5*	64.2	0.5
	criteria	-	±10	±30	n/a	n/a

a Cells marked with “*” are considered outside criteria benchmarks^41^ that are reported in the table, and criteria marked “n/a” were not reported by Emery et al. (2017).

We find strong cold-season agreement with observed Q2 (*r* = 0.9 in February and November) with small errors (ME = 0.8–1.2 g/kg). In the warm season, a drier atmosphere is simulated relative to observations (MB = −0.7 to −1.6 g/kg in May/August), and correlations weaken (*r* = 0.5–0.6).

Wind speeds (WS) are generally negatively biased (MB = −0.3 to −0.5 m/s), remaining near the ±0.5 m/s benchmark. Domain-average RMSE is close to 2 m/s, with a slight exceedance noted in May (RMSE = 2.3 m/s). Correlations are moderate to weak (*r* = 0.2–0.6), with the lowest skill in summer. Wind direction (WD) exhibits the lowest skill. Wintertime errors are near benchmarks (e.g., February MB = 4.9°, ME = 28.4°), whereas larger errors occur in May and August (MB = 7.9° to 10.6°; ME = 67°*), accompanied by low correlations (r = 0.3). November falls between these regimes (MB = 9.4°, ME = 30.6°). Figure S3 shows the corresponding diurnal variation in MB and RMSE for all meteorological variables.

Overall, WRF reproduced temperature and humidity more accurately than wind-related variables, while wind direction exhibited the largest uncertainties, particularly during the warm season. Future work should investigate improved representation of sub-grid-scale meteorological processes, including urban canopy effects, boundary-layer mixing, convective transport, and local circulations, as well as updated land-use datasets and additional observational constraints. These improvements may enhance the simulation of urban meteorology and, consequently, pollutant transport and chemistry.^47^

### 3.2 CMAQ Model Evaluation

CMAQ shows moderate skill for hourly O_3_, with correlations ranging from *r* = 0.4–0.7 and strongest performance in summer (August; Figure 2). The corresponding scatter-density comparisons are shown in Figure S4. Relative to the Emery et al. (2017) benchmarks, hourly O_3_ correlations meet the criterion (*r* > 0.50) only in August. Hourly O_3_ biases range from −13% to +21%, generally within or near the ±15% criterion but exceeding the ±5% goal, while NME values (33–49%) exceed both the 15% goal and 25% criterion. Daily O_3_ MDA8h correlations are lower (*r* = 0.1–0.5), but biases are smaller (NMB = −16% to +7%) and largely within the ±15% criterion, with August approaching the NME goal. Daily mean O_3_ exhibited seasonal bias patterns directionally consistent with those observed for hourly and MDA8 O_3_ (Table S3).

**Figure 2:**
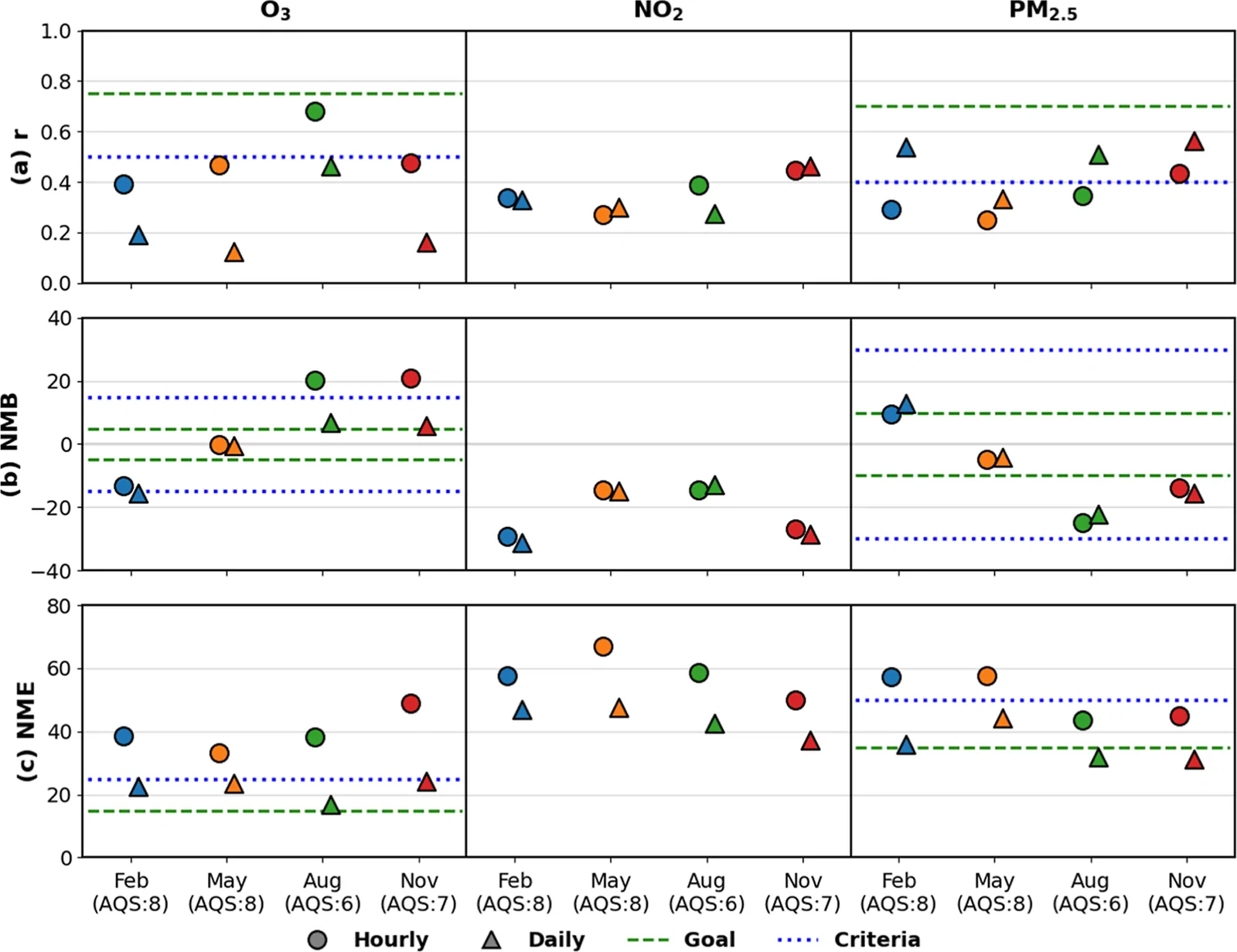
Hourly and daily CMAQ model performance relative to EPA AQS observations of O_3_, NO_2_, and PM_2.5_ in 2017: (a) correlation coefficient (Pearson r), (b) normalized mean bias (NMB), and (c) normalized mean error (NME). Daily O_3_ refers to MDA8h O_3_, and daily NO_2_ and PM_2.5_ refers to the 24 h average. Numbers of comparisons for each evaluation metric vary by season and pollutant (Table S2). Green dashed and blue dotted horizontal lines indicate model performance goal and criteria benchmarks, respectively, based on recommendations from Emery et al. (2017) for MDA8h O_3_ and 24 h PM_2.5_.^40^

NO_2_ exhibits modest correlations (*r* ≈ 0.3–0.5) and a persistent negative bias (NMB = −14% to −29% across months), consistent with known difficulties in resolving near-source NO_*x*_ gradients at kilometer-scale resolution^48^ and potential spatial misallocation of emissions,^49^.^50^ In addition, near-road concentration gradients and localized traffic influences are not well captured, potentially because traffic impacts are diluted even at 1 × 1 km^2^ resolution.^51^ This interpretation is supported by the roadside/non-roadside evaluation (Table S4), which showed substantially larger negative NO_2_ biases at roadside monitoring sites than at non-roadside locations. These limitations are widely reported in high-resolution CTM applications.^48^ ^,^ ^50^ Another potential source of uncertainty is the underlying emissions inventory. Previous evaluations have shown that NEMO reproduces the spatial variability of urban NO_*x*_ emissions in the Washington, D.C. region reasonably well, although satellite comparisons primarily assess spatial patterns rather than emission magnitude.^49^ Therefore, uncertainties in mobile-source NO_*x*_ emission rates may also contribute to the observed negative NO_2_ bias.

The observed O_3_ biases are consistent with uncertainties in NO_*x*_ emissions, VOC emissions, and regional chemical regimes. Because O_3_ was evaluated using the MDA8 metric whereas NO_2_ was evaluated using all hourly observations, an additional analysis was performed using only the hours contributing to the observed daily MDA8 O_3_ metric (Table S5). While hourly NO_2_ exhibited a persistent negative bias across all months (NMB = −14% to −29%), NO_2_ biases during MDA8-contributing hours ranged from +20% in February and +55% in May to approximately unbiased in August (+2%), with only November remaining negatively biased (−9%). These results indicate that the negative NO_2_ bias reported in Figure 2 is primarily associated with non-photochemical periods and that NO_2_ concentrations during the summertime daytime hours most relevant to O_3_ formation are generally represented more accurately by the model.

The relationship between NO_2_ and O_3_ biases is further complicated by nonlinear O_3_ chemistry. In VOC-limited (NO_*x*_-saturated) environments, changes in NO_*x*_ can depress O_3_ concentrations through both titration and radical chemistry, whereas under NO_*x*_-limited conditions O_3_ production generally increases with NO_*x*_ availability. Previous studies in the Baltimore-Washington metropolitan region have demonstrated the coexistence of VOC-limited, transitional, and NO_*x*_-limited chemical regimes that vary spatially and seasonally, reflecting interactions among local emissions, meteorology, and transported pollutants.^21^ These nonlinear chemical responses complicate interpretation of model performance because similar O_3_ biases can arise from multiple sources, including uncertainties in NO_*x*_ emissions, VOC emissions, meteorological conditions, chemical mechanisms, and regional transport. As a result, identifying the specific causes of model error is often more difficult for O_3_ than for primary pollutants, and the relative importance of individual uncertainties may vary seasonally.

Recent studies suggest that volatile chemical products (VCPs), including emissions from consumer and industrial products, can contribute substantially to urban VOC budgets and O_3_ formation For example, Coggon et al.^52^ reported that VCPs account for approximately half of petrochemical VOC emissions in New York City and can make substantial contributions to urban O_3_. This suggests that uncertainties in VCP emissions may contribute to uncertainties in modeled O_3_ formation and secondary pollutant production. Previous evaluations in the Baltimore–Washington region have also shown that CMAQ can underestimate O_3_ production efficiency and radical chemistry, potentially due to incomplete representation of VOC species and uncertainties in chemical mechanisms.^53^

For PM_2.5_, hourly correlations are low to moderate (*r* = 0.3–0.4), while daily correlations improve (*r* = 0.3–0.6) and meet the criterion (*r* > 0.40) in several months. PM_2.5_ biases generally fall within the ±30% criterion (NMB = −24% to +14%), though they do not consistently meet the ±10% goal. Hourly NME (45–63%) exceeds the 50% criterion, whereas daily NME (31–44%) is substantially lower and frequently meets the criterion, with best performance in late summer and fall. The improved daily performance is consistent with previous CMAQ evaluations, where temporal averaging reduces short-term variability and generally improves agreement with observations.^54^

To provide additional context, our model performance is consistent with other high-resolution (≤4 km) CTM studies conducted across the United States (Table S8). A 1.3 km WRF–CMAQ simulation over the Chicago region reported strong correlations for O_3_ and NO_2_ (*r* > 0.6) and weaker performance for PM_2.5_ (*r* ≈ 0.4), along with larger O_3_ biases compared to primary pollutants.^27^ Similarly, a high-resolution CMAQ study in Houston reported strong performance for O_3_ (*r* ≈ 0.8–0.9) and NO_*x*_ (*r* ≈ 0.6–0.7), although discrepancies remained due to uncertainties in emissions, meteorology, and chemistry.^55^ Compared to these studies, our results show slightly lower correlations, particularly for NO_2_ and PM_2.5_, but remain within the expected range for kilometer-scale simulations. These differences likely reflect regional variability in emissions, chemical regimes, and monitoring density, as well as the challenges of resolving sub-grid scale processes in complex urban environments.^47^ ^,^ ^21^ Meteorological uncertainties likely contributed to a portion of the model error. WRF reproduced temperature and humidity relatively better than wind speed and especially wind direction. Overly warm, stagnant conditions in August (positive temperature bias and negative wind speed bias) may contribute to the positive O_3_ bias. With its shorter lifetime, locally emitted NO_2_ would be expected to artificially accumulate under negatively biased wind speed and temperature conditions in the wintertime, but we still observe a negative NO_2_ bias in all months, providing evidence for missing emissions. Since PM_2.5_ is a mix of local and regional,^56^ ^,^ ^26^ it is difficult to link WRF performance with PM_2.5_ bias.

Diurnal performance profiles (Figure S5) show that CMAQ captures the major daily concentration evolutions across seasons, but performance differs by species. For O_3_, both 1 × 1 km^2^ and 4 × 4 km^2^ resolutions reproduce the daytime buildup and afternoon peak, with strongest agreement in summer. Early-morning negative bias and occasional midday positive bias contribute to the overall bias patterns noted in Figure 2. NO_2_ exhibits clear morning and evening peaks but with negative bias, particularly in winter and fall, consistent with the persistent negative bias and known challenges in resolving near-source NO_*x*_ gradients at kilometer-scale resolution.^50^ ^,^ ^51^ Modeled PM_2.5_ shows weaker diurnal variation than observed PM_2.5_, with CMAQ generally underestimating day–night variability, leading to relatively poorer hourly performance compared to daily performance. This reduced variability likely reflects a combination of factors, including uncertainties in the temporal allocation of primary PM_2.5_ emissions, smoothing of localized emission influences within 1 × 1 km^2^ grid cells, uncertainties in boundary-layer evolution that affect nighttime accumulation and daytime dilution, and the substantial contribution of secondary and regionally transported PM_2.5_, which tends to exhibit weaker short-term variability than local primary emissions.^26^ These factors can dampen the modeled diurnal cycle while still allowing reasonable agreement in daily-average concentrations.

### 3.3 Spatial Characterization of CMAQ-Simulated Concentrations at the 1 × 1 km^2^ Domain

The 1 × 1 km^2^ CMAQ results display seasonality and spatial variability of air pollution across Washington, DC (Figures 3; S6). For hourly O_3_, the highest domain-average concentrations occur in August (μ = 34.4 ppb), followed by May (μ = 33.4 pp), while the lowest concentrations are simulated in February (μ = 25.2 pp) and November (μ = 22.2 ppb). Lower O_3_ concentrations during February and November are consistent with weaker photochemical activity, shorter daylight periods and enhanced NO_*x*_ inhibition/titration during cooler seasons. Mean O_3_ outside the urban core (grid cells within a 20 × 20 km^2^ area centered on the model domain) exceeds concentrations near the domain center by 1.3–1.6 ppb (≈4–5%) in May and August, increasing to 2.1–2.5 ppb (≈10–11%) in February and November. In winter and late fall, weaker photochemistry and stronger O_3_ production inhibition by NO_*x*_ within the urban core amplify center–outside contrasts relative to warmer months. Positive bias in urban core NO_2_ levels likely contribute to a negative bias in O_3_ inside-outside urban core gradient (Table S4).

**Figure 3:**
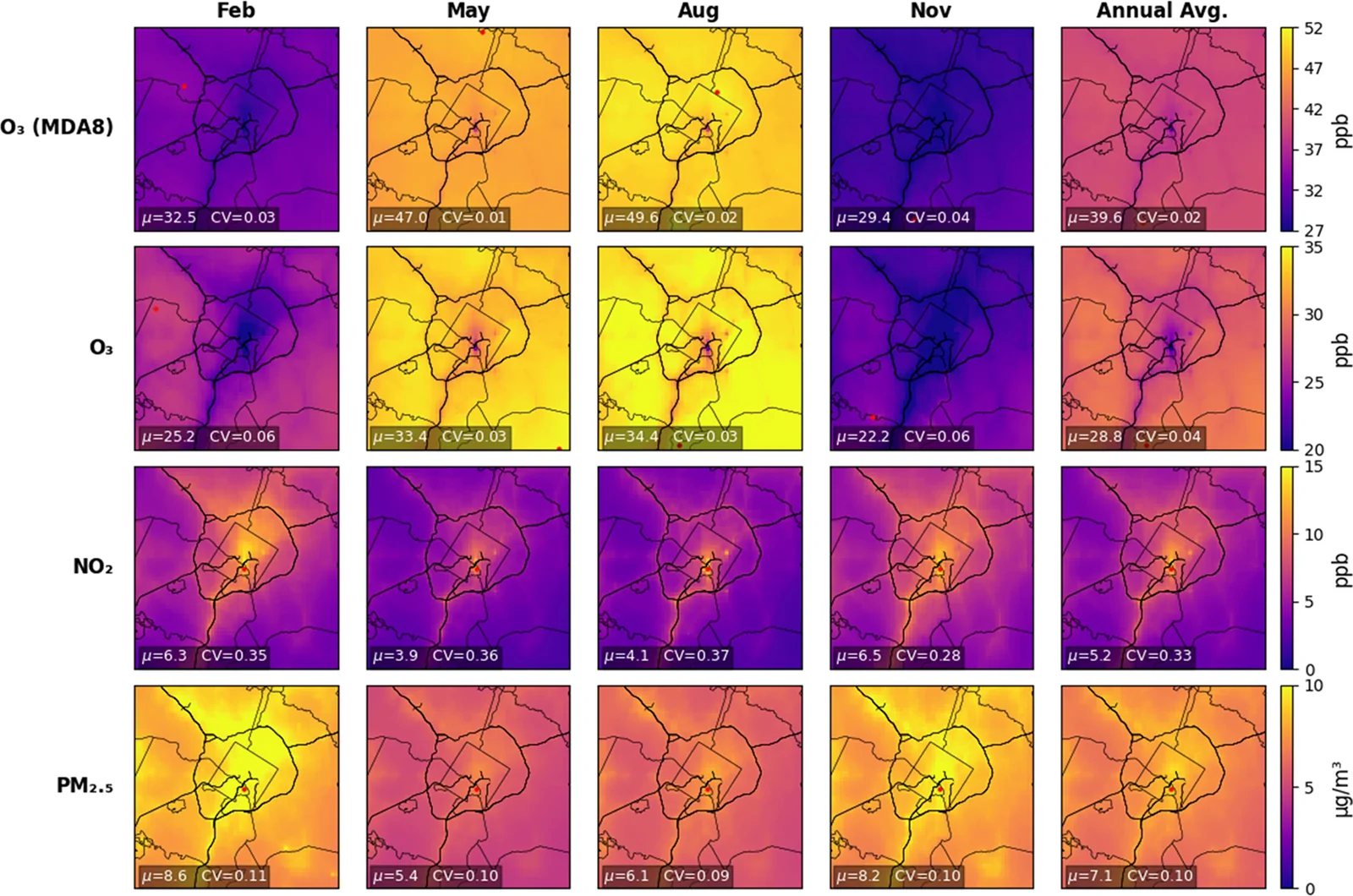
Spatial distributions of simulated O_3_, NO_2_, and PM_2.5_ across the 1 × 1 km^2^ domain (d03) for February, May, August, and November 2017, and the average of all four months. The domain average concentrations (μ) and coefficient of variation (CV = σ/μ) are listed in the lower left corner of each panel, and the location with the highest concentration in each panel is marked with a red dot.

Average monthly NO_2_ varies by nearly *a* factor of 2 across months, with the highest concentrations in November (μ = 6.5 ppb) and lowest in May (μ = 3.9 ppb), reflecting reduced photochemical loss and shallow wintertime boundary layers in cooler temperatures and more oxidation and vertical mixing in warmer periods. Additionally, NO_*x*_ emissions are 5.1% higher in November than in May (Figure S7). NO_2_ exhibits strong spatial gradients; in all seasons, the highest NO_2_ concentrations occur along major road networks, including I-495, I-95, I-66, US-50, and the Baltimore-Washington Parkway, consistent with dominant contributions from mobile-source NO_*x*_ emissions.

PM_2.5_ displays less spatial variability in the domain compared to NO_2_, as indicated by consistently smaller domain-wide coefficient of variation (CV), with only modest enhancement in PM_2.5_ near the urban core and along other major highways. The highest concentrations occur in February (μ = 8.6 μg/m^3^), consistent with stagnant meteorology, greater primary PM_2.5_ emissions from sources such as residential combustion (μ = 0.04 g/s; Figure S7) and reduced atmospheric mixing. PM_2.5_ concentrations are lowest in May (μ = 5.4 μg/m^3^). The relatively smooth spatial patterns reflect the dominant contribution of secondary and regionally transported PM (e.g., sulfate and SOA), which reduces local variability compared to primary pollutants.^56^ ^,^ ^26^

NO_2_ exhibits the strongest spatial gradients, with an annual CV ≈ 0.33 that is more than eight times that of O_3_ (CV ≈ 0.04) and more than three times that of PM_2.5_ (CV ≈ 0.1). PM_2.5_ exhibits intermediate spatial variability, reflecting contributions from both local and regional sources, whereas O_3_ shows the smoothest spatial patterns across the domain. Annual mean NO_2_ concentrations in central and northeastern neighborhoods are roughly twice those in the least polluted areas. PM_2.5_ concentrations are elevated in downtown and northeastern neighborhoods and reduced along the northern and western edges of the city. Annual average O_3_ concentrations are lowest in traffic-dominated central and northeastern neighborhoods, where NO_*x*_ inhibition/titration is strongest, and highest along the less densely developed western and southeastern edges of the District.

#### 3.3.1 Comparing Model Performance at 1 km and 4 km Resolution

Our modeling framework allows assessment of how refining horizontal grid spacing from 4 × 4 km^2^ to 1 × 1 km^2^ alters simulated exposure on D.C.’s neighborhood clusters. Most clusters are covered by parts of 5–8 1 × 1 km^2^ grid cells, but only a single 4 × 4 km^2^ grid cell (Figure S8).

Compared to the broader domain, annual average concentrations within D.C. are generally lower for NO_2_ (by 6%) and PM_2.5_ (3%) and slightly higher for O_3_ (3%) (Figure 4). Monthly average concentrations simulated by the 1 × 1 km^2^ framework are presented in Figure S9, illustrating the seasonal variability underlying these annual-average differences. Because chemistry is treated consistently across nested domains, differences between the 4 × 4 km^2^ and 1 × 1 km^2^ simulations reflect both the inherent ability of finer grids to resolve sharper spatial gradients through reduced averaging volumes and resolution-dependent changes in emissions allocation, meteorological processes and chemical transformations. These meteorological influences include boundary-layer mixing and the representation of horizontal transport, radiation, cloud processes, and precipitation, all of which can affect pollutant concentrations and exposure estimates.^57^ ^,^ ^58^ As a result, this comparison emphasizes how grid resolution reshapes pollutant fields rather than serving as a strict numerical resolution sensitivity test.^42^ ^,^ ^59^

**Figure 4:**
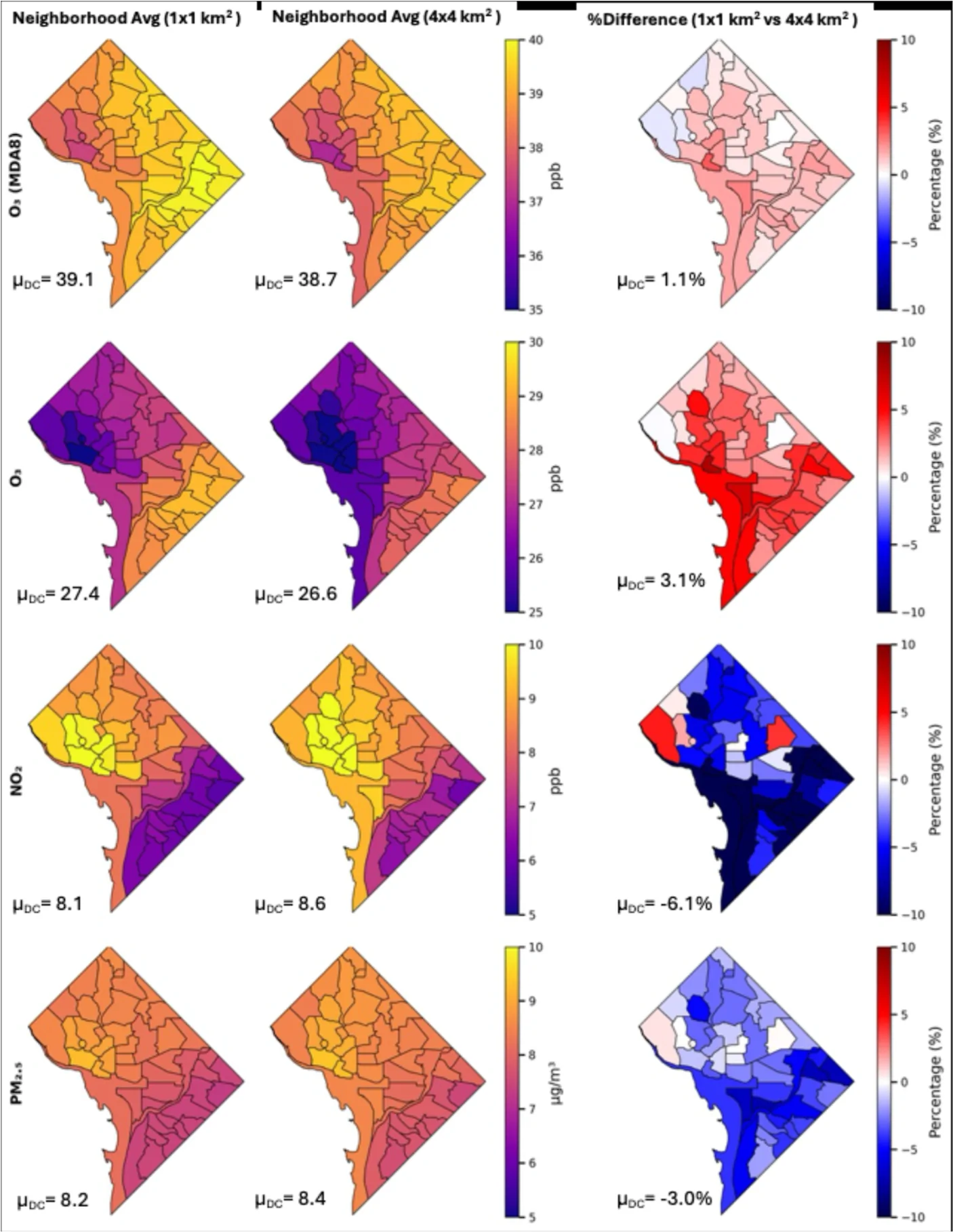
Area-weighted annual average MDA8h O_3_, O_3_, NO_2_, and PM_2.5_ within Washington, DC, simulated at 1 × 1 km^2^ and 4 × 4 km^2^ resolution, and percent differences between the 1 × 1 km^2^ and 4 × 4 km^2^ simulations.

Model performance differences between resolutions are pollutant and season-dependent (Table S7; Figure S5). For O_3_, differences in spatial–temporal correlation are small (|Δ*r*| < 0.1), although the 1 × 1 km^2^ simulation yields slightly higher seasonal mean error and RMSE in some months. For NO_2_, the 1 × 1 km^2^ simulation reduces the magnitude of mean bias in most seasons, but this improvement is accompanied by higher RMSE and lower correlations relative to the 4×4 km^2^ simulation. These results indicate that, although finer resolution can improve mean concentration estimates by better resolving near-source emissions, it does not necessarily improve the model’s ability to capture the full spatiotemporal variability of NO_2_. The degraded RMSE and correlation may reflect a combination of uncertain near-road gradients and temporal misalignment of observed and modeled morning and evening peaks at individual monitoring sites. For PM_2.5_, the 1 × 1 km^2^ resolution improves RMSE and correlation during spring through fall, while winter performance degrades consistent with PM_2.5_’s weaker sensitivity to model resolution and greater regional contribution.

Longitudinally averaged concentration ratios across the finest model domain (Figure S11) show that 1 × 1 km^2^/4 × 4 km^2^ NO_2_ ratios are mostly below unity (≈0.92–0.97), implying that the coarser 4 × 4 km^2^ grid distributes NO_2_ emissions over a larger grid cells and produces different spatial gradients than the 1 × 1 km^2^ simulation. The ratios vary across the domain, with 1 × 1 km^2^ NO_2_ concentrations exceeding 4 × 4 km^2^ concentrations mainly near the urban core and major transportation corridors. In contrast, PM_2.5_ ratios remain closer to unity, consistent with its larger regional component and weaker sensitivity to horizontal grid refinement. O_3_ is elevated outside the urban core where NO_*x*_ inhibition/titration is weaker, and average O_3_ ratios are slightly above 1 (≈1.02–1.03) across much of the domain, particularly outside the urban core where NO_*x*_ inhibition and O_3_ titration are weaker.

Overall similar performance in CMAQ output for higher resolution domains is generally consistent with prior studies that report evaluation statistics at multiple resolutions (Table S8).^27^ ^,^ ^55^ Still, our finding of overall increased O_3_ and decreased NO_2_ concentrations in the higher resolution domain differs from a similar effort over Chicago, IL,^27^ which found downtown decreases and suburban increases in O_3_ and vice versa for NO_2_. In the D.C. area, there is evidence that higher resolution meteorology inputs better resolve the Chesapeake Bay/land breeze pattern leading to higher modeled O_3_, but, because our smallest domain does not include the Bay, this is not a major contributor to our results.^47^ Recent studies have also shown that increasing the spatial resolution of air quality modeling can reveal substantially greater spatial variability in pollutant exposure and associated disparities than coarser-resolution analyses, particularly when combined with high-resolution population data.^60^ ^,^ ^46^ Although O_3_ is generally considered more spatially homogeneous than primary pollutants such as NO_2_, our results demonstrate that high-resolution modeling can reveal important neighborhood-scale differences in O_3_ responses to emission changes.

The complementary O_3_ and NO_2_ responses observed across grid resolutions are consistent with the rapid photochemical cycling between these species. Future work could evaluate total oxidant concentrations (O_*x*_ = O_3_ + NO_2_), which may provide a more stable measure of atmospheric oxidative capacity and reduce sensitivity to resolution-dependent partitioning between O_3_ and NO_2_. Such an approach may also be relevant for exposure and health impact assessments that seek to characterize the combined effects of photochemical oxidants.^61^

#### 3.3.2 Population-Weighted Exposure and Resolution-Dependent Exposure Distributions

While spatial concentration patterns reveal resolution-dependent differences in pollutant gradients, their implications for public health depend on how pollutant levels intersect with population density. Refining grid resolution from 4 × 4 km^2^ to 1 × 1 km^2^ consistently increased PWE for both O_3_ metrics (Figure 5 shows relative differences; Figure S12 shows absolute PWE). Median PWE differences for MDA8 O_3_ ranged from about +0.5% to +1.5%. These increases are consistent with the spatial distribution of O_3_ differences shown in Figure 4, where most neighborhood clusters exhibit higher O_3_ concentrations in the 1 × 1 km^2^ domain than in the 4 × 4 km^2^ domain. The relatively few clusters with lower O_3_ concentrations at 1 × 1 km^2^ generally occur in less populated areas (Figure S2), resulting in a net increase in population-weighted exposure across the District. Notably, the largest O_3_ PWE differences occurred during cooler months, reflecting the greater influence of local NO_*x*_ gradients and O_3_ titration under lower-photochemical conditions, which increases the sensitivity of O_3_ concentrations to grid-resolution effects.

**Figure 5:**
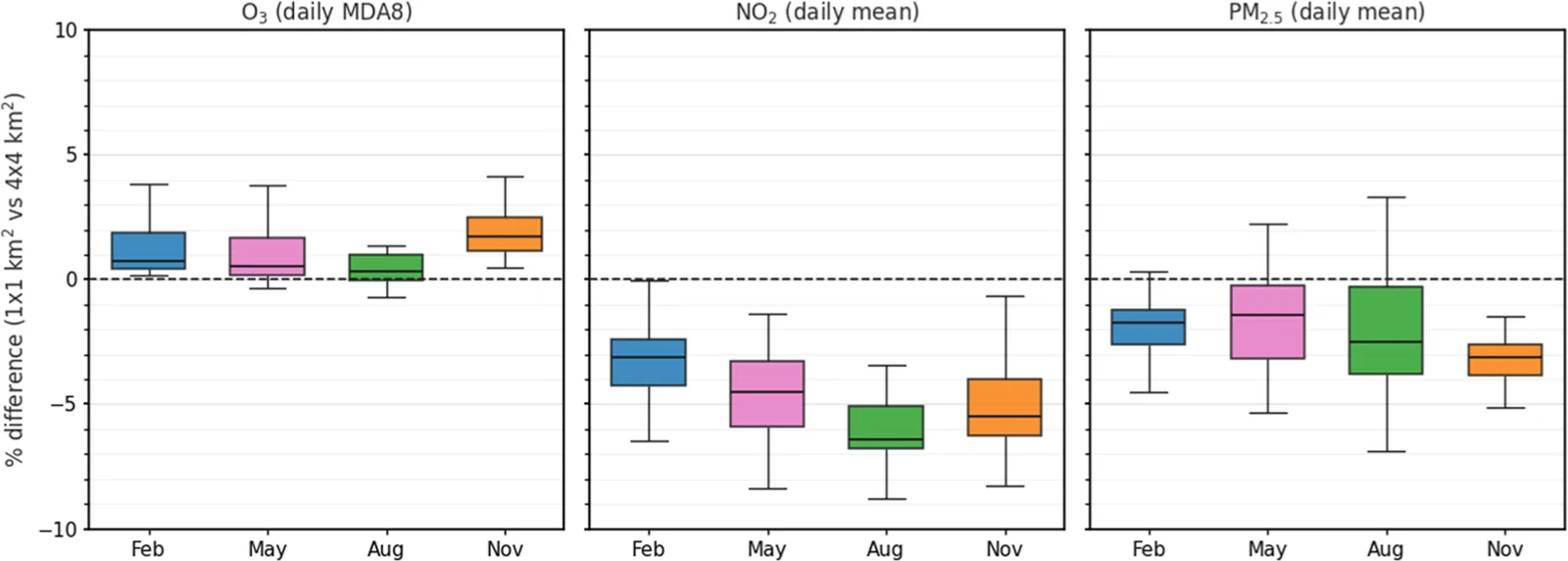
Percent differences in daily population-weighted exposure between 1 × 1 km^2^ and 4 × 4 km^2^ CMAQ simulations over Washington, D.C. for MDA8h O_3_, NO_2_, and PM_2.5_ in each month. Daily metrics summarized in the box plots are based on 28 days in February, 31 in May, 31 in August, and 30 in November.

In contrast to O_3_, NO_2_ and PM_2.5_ showed consistent decreases in PWE at finer resolution, with median differences of −3% to −6% for NO_2_ and −1.5% to −3% for PM_2.5_ across all months. At 4 × 4 km^2^ resolution, high-concentration roadway corridors are spatially diluted. Comparison of the aggregated 1 × 1 km^2^ and native 4 × 4 km^2^ concentration fields across the full modeling domain (Figure S10) indicates that the 4 × 4 km^2^ simulation produces systematically higher NO_2_ and PM_2.5_ concentrations over much of the domain. This pattern reflects differences in how emissions, transport, mixing, and chemistry are represented across resolutions, resulting in broader spatial redistribution of pollutant concentrations in the coarser simulation. Consequently, population-weighted exposures for NO_2_ and PM_2.5_ are generally lower in the 1 × 1 km^2^ simulation.

Spatial concentration variability is particularly relevant in Washington, D.C., which exhibits well-documented socioeconomic and racial disparities and where fine-scale pollutant gradients may contribute to differences in population exposure.^8^ ^,^ ^9^ ^,^ ^62^ Although this study does not explicitly evaluate environmental justice outcomes, the 1 × 1 km^2^ framework provides a spatial scale more appropriate for investigating neighborhood-level exposure variability than coarser-resolution simulations. One limitation of this analysis is that PWE were calculated at the neighborhood cluster level, which may not capture sub-neighborhood variability in population and pollutant concentrations. This is especially relevant for near-road pollutants like NO_2_, which decline rapidly within a few hundred meters of major roadways.^63^ As a result, averaging across clusters may smooth important gradients. Incorporating more spatially detailed population information in future analyses could help better represent intra-neighborhood variability and its implications for environmental justice. These findings suggest that kilometer-scale modeling provides additional information for evaluating air quality management strategies and population exposure beyond what can be obtained from coarser-resolution simulations.

#### 3.3.3 Chemical Drivers of O_3_ Formation

Tropospheric O_3_ production is driven by photochemical reactions of VOCs and NO_*x*_, where the balance between these precursors dictates whether the atmosphere behaves as VOC-limited, NO_*x*_-limited, or transitional in its potential to generate O_3_.^64^ ^,^ ^65^

Although no single VOC/NO_*x*_ (or HCHO/NO_2_) threshold uniquely defines O_3_ chemical regimes, ranges are often used for interpretation. Low ratios (approximately <1) are generally associated with VOC-limited conditions, whereas high ratios (approximately >2 for column measurements, or >7 for surface-based estimates in urban settings) indicate NO_*x*_-limited chemistry. Intermediate values are typically interpreted as transitional between regimes.^66^ ^,^ ^67^ When VOC/NO_*x*_ ratios are low, excess NO scavenges peroxyl radicals (HO_2_, RO_2_) and suppresses ozone formation, whereas higher VOC availability supports radical propagation and can accelerate O_3_ production under sufficient photolysis.^68^

Because no single indicator uniquely defines O_3_ sensitivity, multiple metrics have been proposed, including VOC/NO_*x*_, HCHO/NO_2_, and H_2_O_2_/HNO_3_ ratios.^66^ ^,^ ^67^ In this study, we primarily use the daytime surface VOC/NO_*x*_ ratio, calculated from CMAQ surface-layer concentrations, where VOC concentrations were calculated as the sum of major reactive VOC species available in the CB6r5_ae7_aq chemical mechanism, including representative alkanes, alkenes, aromatics, aldehydes, and biogenic hydrocarbons (Table S9). Individual species were converted from mixing ratios (ppbv) to carbon mixing ratios (ppbC) by multiplying each species by its molecular carbon number prior to summation. This carbon-weighted VOC/NO_*x*_ metric provides a physically intuitive representation of local precursor availability (Figures S13 and S15). By contrast, HCHO/NO_2_ is commonly derived from satellite column observations (e.g., TROPOMI) and is widely used to characterize the spatial distribution of O_3_ chemical regimes where surface observations are limited.^66^ To provide an additional and widely used O_3_-sensitivity indicator, we also present daytime HCHO/NO_2_ column ratios (Figures S14 and S15). In all months, O_3_ indicates a morning minimum during peak NO_*x*_ influence (early commute hours, shallow boundary layer and limited downward mixing of O_3_ from the residual layer), then rises to a midday/early-afternoon maximum under stronger photochemical production (Figure 6). The VOC/NO_*x*_ ratio follows a similar (but opposite) diurnal evolution, remaining lowest in the morning and increasing toward midday, consistent with reduced NO_*x*_ inhibition/titration and enhanced radical-driven chemistry during peak sunlight. Seasonally, VOC/NO_*x*_ ratios increase from winter to summer VOC/NO_*x*_ ratios were 0.9 in February and 4 in August across the District, in alignment with previous work showing stronger photochemical activity and greater VOC availability during warmer months.^25^

**Figure 6:**
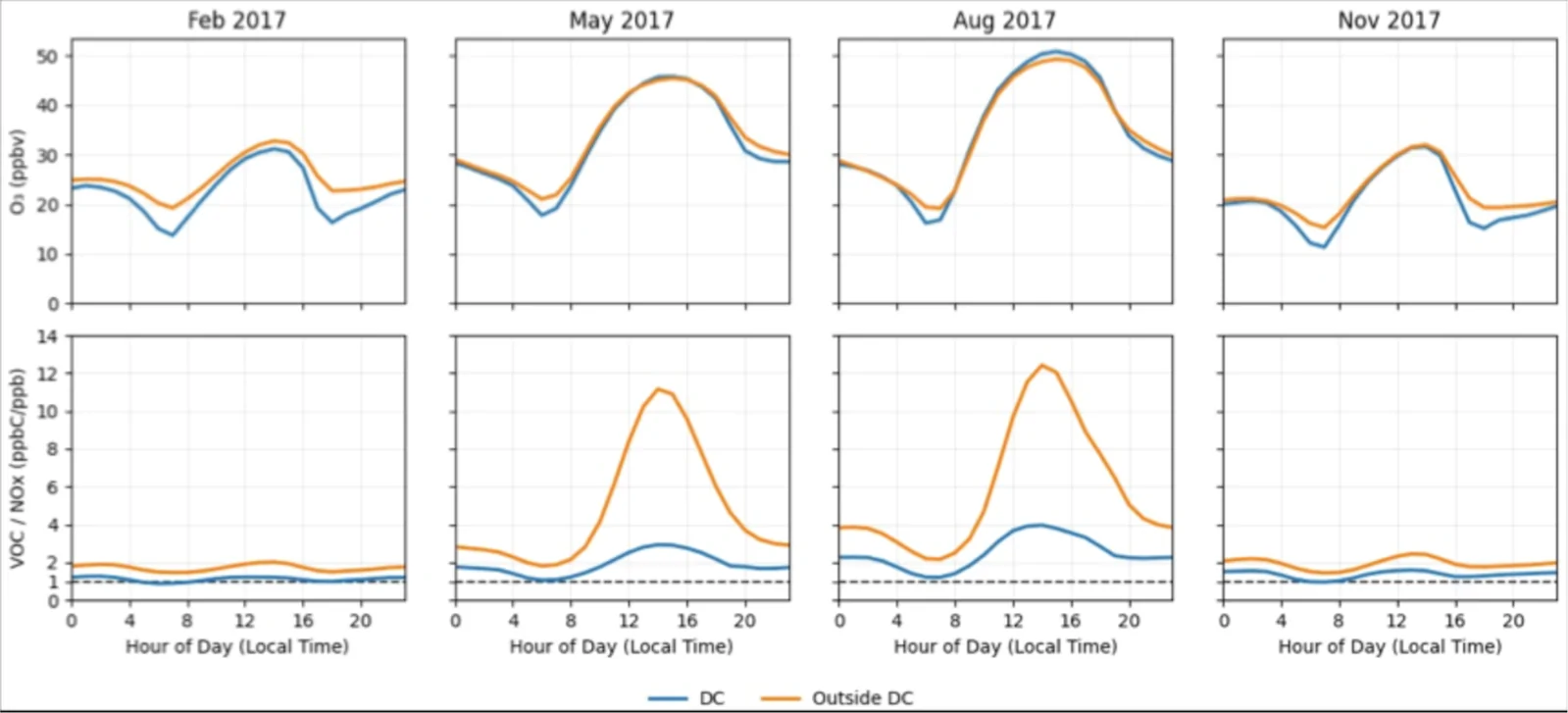
Diurnal Variation of O_3_ and VOC/NO_*x*_ within Washington, D.C. and outside the urban core.

Compared to within Washington D.C., VOC/NO_*x*_ outside the urban core are larger by *a* factor of about 2 in winter and up to 3.6 in summer (VOC/NO_*x*_ of 1.5 in February to 12.5 in August), indicating a substantially greater sensitivity to NO_*x*_ outside the urban core. Urban-suburban differences are largest in May and August, with persistently lower VOC/NO_*x*_ ratios within the urban core and along major transportation corridors, indicating a VOC limited environment, while higher ratios outside the core reflect greater VOC availability (Figures S13 and S15). Similar spatial patterns are evident in the HCHO/NO_2_ ratios (Figures S14 and S15), which show lower values within Washington, D.C. and along major transportation corridors and higher values outside the urban core, supporting the interpretation derived from the VOC/NO_*x*_ analysis.

Together, the VOC/NO_*x*_ and HCHO/NO_2_ diagnostics suggest that the local photochemical environment within Washington, D.C. is generally VOC-limited to transitional, particularly along major transportation corridors where NO_*x*_ concentrations remain elevated.^69^ During summer, portions of the urban core move closer to transitional conditions, suggesting that both VOC and NO_*x*_ emission reductions may influence O_3_ formation. Outside the urban core, higher VOC/NO_*x*_ and HCHO/NO_2_ ratios indicate a shift toward more NO_*x*_-sensitive conditions during warm-season periods. These findings are consistent with the spatial and seasonal variability in O_3_ observed across the modeling domain. Our findings are broadly consistent with previous observational and modeling studies in the Baltimore-Washington region that reported VOC-limited to transitional conditions in the urban core and increasingly NO_*x*_-sensitive outside the city.^21^ ^,^ ^53^ Here, we extend these findings by resolving neighborhood-scale variability in O_3_ chemical regimes at 1 × 1 km^2^ resolution, providing spatial detail along transportation corridors and urban-core neighborhoods where NO_*x*_ gradients are strongest (Figure S16). The comparison between resolution shows that coarser-resolution models or satellite-derived products may not fully capture neighborhood-scale variability in O_3_ chemical sensitivity.^23^ ^,^ ^27^

However, O_3_ concentrations in the region are influenced by both local photochemistry and regional transport of O_3_ and its precursors. Therefore, these indicators should be interpreted as describing local chemical sensitivity rather than the complete O_3_ sensitivity to regional emissions. The results provide insight into neighborhood-scale variability in photochemical sensitivity across Washington, D.C. This spatial heterogeneity helps explain why similar changes in precursor emissions may produce different local O_3_ responses and provides additional context for interpreting the population exposure differences presented in the previous section.

### 3.4 Limitation

Although the framework provides spatial detail sufficient for resolving neighborhood-scale pollutant gradients, PWE, and O_3_ chemical sensitivity, several limitations should be considered when interpreting the results. The 1 × 1 km^2^ grid spacing remains insufficient to explicitly resolve sub-grid processes such as roadway-scale emissions, street-canyon circulation, building-induced turbulence, and near-road dispersion. Consequently, concentration gradients near major roadways remain partially unresolved, potentially contributing to underestimation of peak NO_2_ concentrations and smoothing of local pollutant variability.^70^ ^,^ ^71^

Meteorological uncertainties directly affect pollutant transport and chemical regimes. In this study, simulations were conducted for four representative months (February, May, August, and November) due to computational constraints, which may not fully capture interannual variability or transitional seasonal dynamics. While this approach captures seasonal variability, it may not fully represent inter-month variability or episodic events. In addition, the WRF configuration did not employ an urban canopy parameterization and retained the Kain-Fritsch cumulus parameterization for the 1 × 1 km^2^ domain. Alternative urban-focused physics configurations, including urban canopy parameterizations and convection-permitting approaches, may improve the representation of urban meteorology and pollutant transport. Finally, the use of offline coupling between WRF and CMAQ, rather than two-way coupled simulations, may limit feedback processes between chemistry and meteorology that could improve model performance.^72^

Emissions inventories include uncertainties in temporal allocation, mobile-source activity, and small-area sources that influence neighborhood-scale gradients. The negative NO_2_ bias suggests that emissions may exhibit a similar negative bias.^49^ ^,^ ^73^ Prior work in the region also indicates potential underestimation of O_3_ production efficiency due to VOC and chemical mechanism uncertainties, suggesting that both NO_*x*_ and VOC emissions may be biased low.^52^ However, the combined implications of these biases for simulated pollutant concentrations are difficult to disentangle, given the nonlinear interactions governing O_3_ formation. Further work is needed to better constrain precursor emissions and chemical processes in urban environments.

Finally, uncertainties associated with the chemical mechanism and emissions representation within CMAQ contribute to seasonal biases in O_3_ and PM_2.5_. In addition, the selected WRF-CMAQ configuration, including the vertical grid structure, may influence the representation of boundary-layer mixing and pollutant transport. These limitations are consistent with previous high-resolution urban modeling studies but should be considered when interpreting small-scale variability.^55^ ^,^ ^74^ ^,^ ^75^ Our model evaluation was limited to available monitors within the domain. Increasing observations, particularly in under-monitored urban areas, would improve evaluation robustness.

## 4 Conclusions

This study demonstrates that high-resolution (1 × 1 km^2^) air quality modeling improves the characterization of intra-urban spatial and temporal variability in Washington, D.C. over monitor data and coarser model grids. We demonstrated the value of kilometer-scale modeling for resolving neighborhood-scale spatial variability in pollutant concentrations, PWE, and O_3_ chemical sensitivity. The finer-resolution framework preserved sharper near-road concentration gradients and reduced spatial averaging inherent to coarser grids, providing additional spatial detail for interpreting urban air quality patterns. While the model captures seasonal and diurnal NO_2_ and O_3_ trends reasonably well, persistent NO_2_ underestimation and uncertainties in O_3_ indicate that urban NO_*x*_ emissions may be underestimated or spatially diluted, while VOC emissions be incompletely represented.

Together, these biases can shift the modeled chemical regime toward lower precursor availability, leading to suppressed NO_2_ concentrations and misrepresentation of O_3_ formation, thereby complicating interpretation of absolute concentration levels and chemical sensitivity. Importantly, increasing the horizontal resolution altered population-weighted NO_2_ and PM_2.5_ exposure estimates through differences in the representation of emissions, transport, mixing, and chemistry, resulting in systematically different concentration fields between the 1 × 1 km^2^ and 4 × 4 km^2^ simulations. This finding is consistent with previous high-resolution urban CTM studies demonstrating that increasing model resolution can substantially influence exposure estimates by resolving finer-scale spatial variability.^27^ Finally, the analysis of VOC/NO_*x*_ regimes suggests that O_3_ formation in the urban core transitions from predominantly VOC-limited toward transitional conditions during the hottest part of day in the summer, indicating that both VOC and NO_*x*_ emissions may influence peak O_3_ concentrations. However, these results should be interpreted with caution because O_3_ concentrations in the region are influenced by uncertainties in emissions, meteorology, atmospheric transport, and chemical processes, as well as regional transport of O_3_ and its precursors.

Nevertheless, the analysis provides a new neighborhood-scale characterization of O_3_ chemical sensitivity across Washington, D.C., identifying locations where local photochemical environments differ and providing additional context for interpreting spatial variability in population exposure. These findings demonstrate that kilometer-scale CTM simulations provide information beyond conventional regional modeling by resolving neighborhood-scale pollutant gradients, exposure differences, and photochemical variability that are directly relevant to urban air quality assessment. For Washington, D.C., where O_3_ remains the primary air pollutant of regulatory concern and substantial spatial disparities in air pollution exposure have been documented, these results highlight the importance of considering neighborhood-scale variability when interpreting population exposure and evaluating future air quality management strategies. Although the present study does not evaluate emission-control scenarios or environmental justice outcomes directly, the modeling framework provides a foundation for future work investigating regulatory applications, exposure disparities, and health impacts at neighborhood scales.

## Supplementary Material



## Data Availability

The processed meteorological evaluation products, aggregated CMAQ outputs, and population-weighted exposure datasets supporting this study are publicly available through the George Mason University Dataverse.^76^ Raw WRF and CMAQ model outputs are not included because of their large size but are available from the corresponding author upon reasonable request.

## Notes

The Supporting Information includes locations of EPA AQS monitoring sites and Washington, D.C. neighborhood clusters; definitions of model performance metrics; meteorological and CMAQ evaluation results; comparisons of 1 × 1 km^2^ and 4 × 4 km^2^ simulations; monthly emissions and concentration distributions; PWE analyses; VOC/NO_*x*_ species definitions and O_3_ chemical-regime diagnostics; and additional figures and tables supporting the main text (PDF)
