# Supplementary material for: Neighborhood-Scale O3, NO2, and PM2.5 in Washington, D.C. from a 1 × 1 km2 WRF–SMOKE–CMAQ Framework
Source: ACS EST Air. 2026 Sep 4;3(10):2640–53. doi: 10.1021/acsestair.6c00155 (PMC13660353; doi:10.1021/acsestair.6c00155)
Supplement: Supplementary file 1 [file ea6c00155_si_001.pdf]

Supplementary Material:

Neighborhood-Scale O_3, NO_2, and $PM_{2.5}$ in Washington, D.C. from a 1x1 km^2 WRF–SMOKE–CMAQ Framework

Hoda Hallaji[a*], Siqi Ma [b], Daniel Q. Tong[b], Lucas R.F. Henneman[a]

[a]Civil, Environmental, and Infrastructure Engineering Department, George Mason University, Fairfax, VA 22030, USA

[b]Atmospheric, Oceanic & Earth Sciences Department, George Mason University, Fairfax, VA 22030, USA

[*]Corresponding author: Hoda Hallaji (hhallaji@gmu.edu)

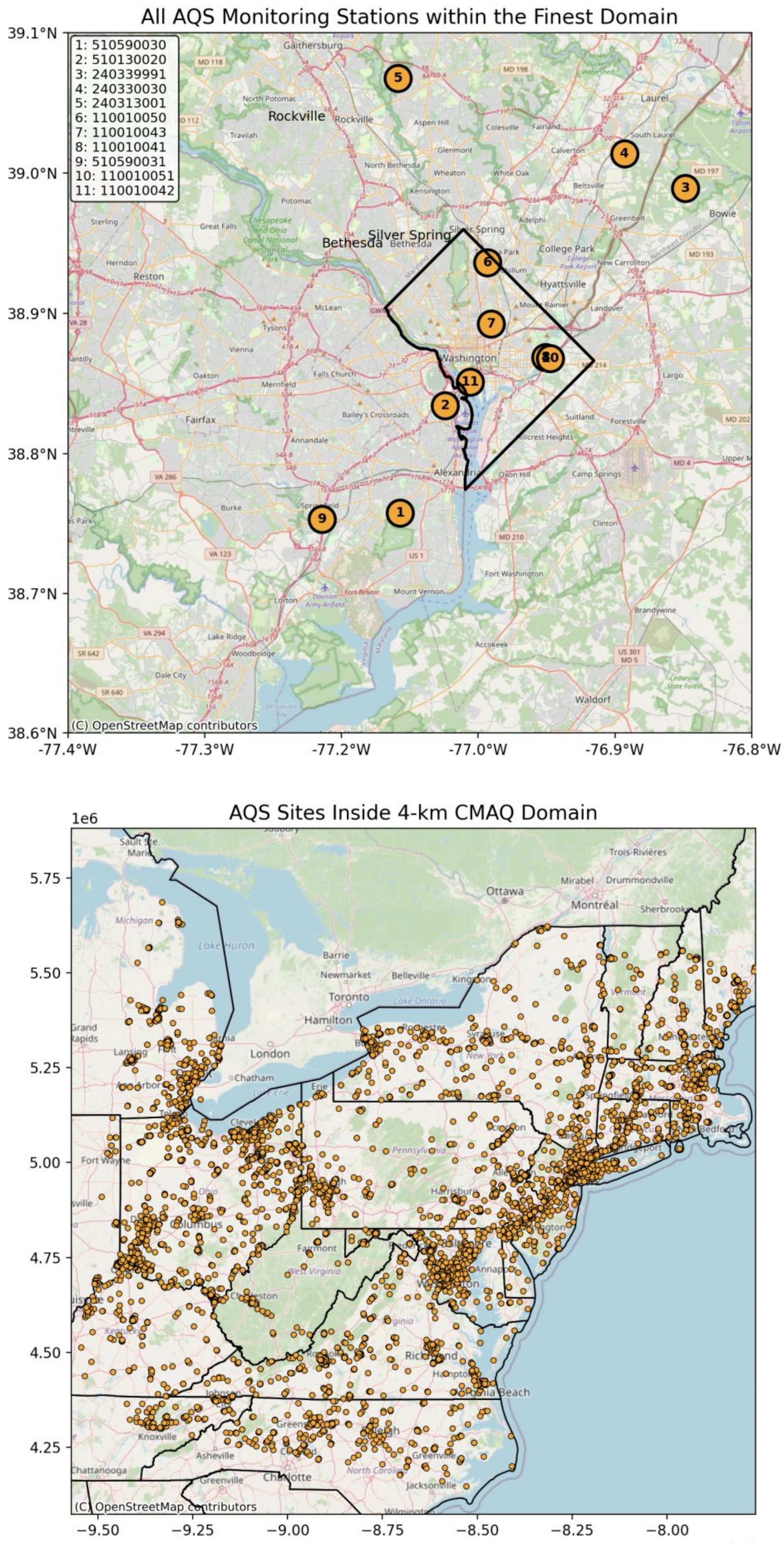

Figure S1. Location of AQS Stations over the 1×1 km^2 and 4×4 km^2 Domain

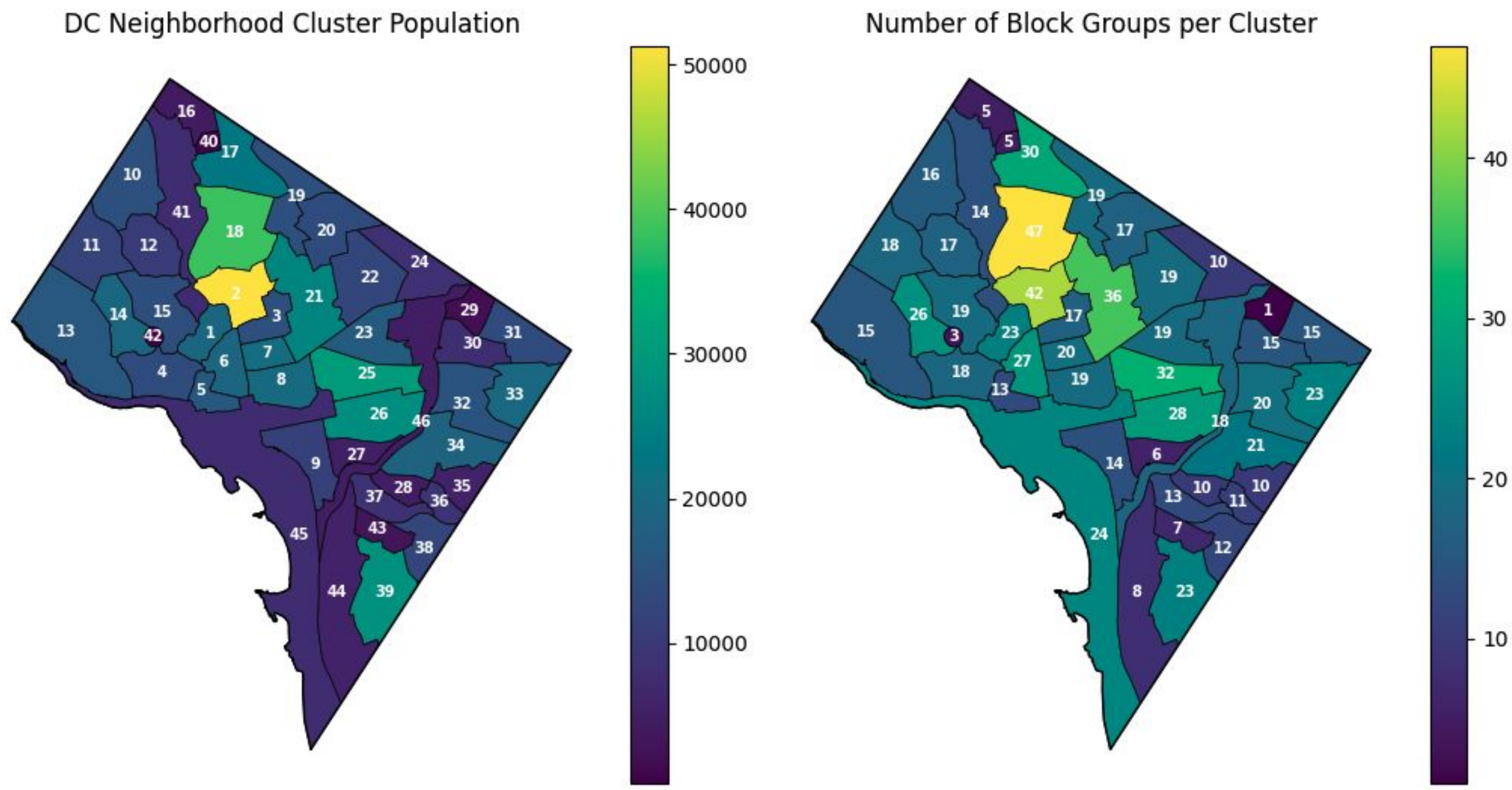

Figure S2. Spatial distribution of Washington, D.C. neighborhood clusters showing (left) total cluster population and (right) the number of census block groups intersecting each cluster. For example, Neighborhood Cluster 2, with an estimated population of 51,337, is composed of 42 intersecting census block groups.

Table S1. Definition of performance metrics used in this study. Subscript j represents the pairing of N observations (O) and predictions (P) by site and time.

Metrics	Definition
Mean ($\boldsymbol{\mu}$)	$\mu = \frac{1}{n}\sum_{j=1}^{n} P_j$
Spatial Standard Deviation (σ)	$\sigma = \sqrt{\frac{1}{n}\sum_{j=1}^{n} (P_j - \mu)^2}$
Coefficient of Variation	$CV = \frac{\sigma}{\mu}$
Mean Bias (*MB*)	$MB = \frac{1}{n}\sum_{1}^{n} (P_j - O_j)$
Mean Error (*ME*)	$ME = \frac{1}{n}\sum_{1}^{n} \lvert P_j - O_j \rvert$
Root Mean Squared Error (*RMSE*)	$RMSE = \sqrt{\sum_{1}^{n} \frac{(P_j - O_j)^2}{n}}$
Normalized Mean Bias (*NMB*)	$NMB = \frac{\sum_1^n (P_j - O_j)}{\sum_1^n O_j}$
Normalized Mean Error (*NME*)	$NME = \frac{\sum_1^n \lvert P_j - O_j \rvert}{\sum_1^n O_j}$
Correlation Coefficient (*r*)	$r = \frac{\sum[(P_j - \overline{P}) \times (O_j - \overline{O})]}{\sqrt{\sum (P_j - \overline{P})^2 \times \sum (O_j - \overline{O})^2}} \times 100$

Table S2. EPA AQS monitoring sites within the 1×1 km^2 (d03) domain and the pollutants measured (O_3, NO_2, $PM_{2.5}$) during each evaluation month

	SiteID	Feb	May	Aug	Nov
1	510590030	$PM_{2.5}$	O_3 - $PM_{2.5}$	O_3 - $PM_{2.5}$	O_3 - $PM_{2.5}$
2	510130020	NO_2	O_3 - NO_2	O_3 - NO_2	O_3 - NO_2
3	240339991	O_3	O_3	O_3	O_3
4	240330030	O_3 - NO_2 - $PM_{2.5}$	O_3 - NO_2 - $PM_{2.5}$	O_3 - NO_2 - $PM_{2.5}$	O_3 - NO_2 - $PM_{2.5}$
5	240313001	$PM_{2.5}$	O_3 - $PM_{2.5}$	O_3 - $PM_{2.5}$	O_3 - $PM_{2.5}$
6	110010050	O_3 - NO_2	O_3 - NO_2	O_3 - NO_2	O_3 - NO_2
7	110010043	O_3 - NO_2 - $PM_{2.5}$	O_3 - NO_2 - $PM_{2.5}$	O_3 - NO_2 - $PM_{2.5}$	O_3 - NO_2 - $PM_{2.5}$
8	110010041	O_3 - NO_2 - $PM_{2.5}$	O_3 - NO_2 - $PM_{2.5}$	O_3 - NO_2 - $PM_{2.5}$	O_3 - NO_2 - $PM_{2.5}$
9	510590031	NO_2 - $PM_{2.5}$	NO_2 - $PM_{2.5}$	NO_2	NO_2 - $PM_{2.5}$
10	110010051	NO_2 - $PM_{2.5}$	NO_2 - $PM_{2.5}$	NO_2 - $PM_{2.5}$	NO_2 - $PM_{2.5}$
11	110010042	$PM_{2.5}$	$PM_{2.5}$	_	_

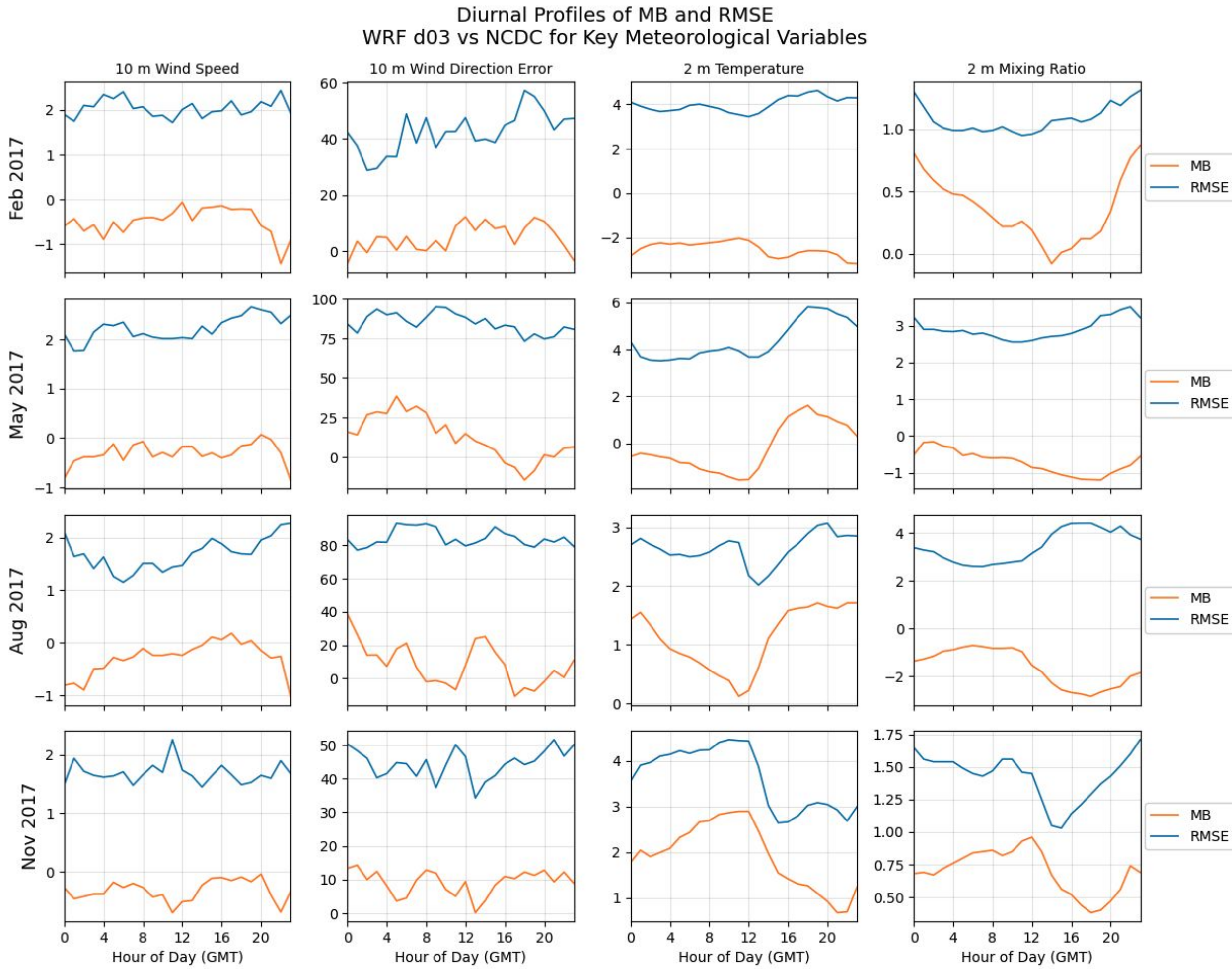

Figure S3. Diurnal profiles of MB and RMSE for meteorological variables averaged over all 7 NCDC stations and their containing grid cells in the 1×1 km² domain

Table S3: CMAQ Performance Metrics for Simulated 1×1 km^2 (d03) Pollutants as Compared to Environmental Protection Agency Air Quality System Station Observations

Pollutant	Month	MB	NMB	RMSE	r	Number of AQS
**O_3**	Feb	-3.4	-13.2	12.5	0.4*	5
	May	-0.1	-0.3	13.6	0.5	8
	Aug	5.6	20.2*	13.5	0.7	8
	Nov	3.5	20.8*	10.6	0.5	8
**Daily O_3**	Feb	-3.2	-12.5	8.9	0.3	5
	May	-0.1	-0.46	9.3	0.02	8
	Aug	5.6	20.3	10.2	0.1	8
	Nov	4.1	25.2	8.0	0.2	8
**NO_2**	Feb	-4.5	-29.1	12.7	0.3	7
	May	-1.2	-14.4	8.3	0.3	7
	Aug	-1.4	-14.7	7.7	0.4	7
	Nov	-3.7	-27.0	9.3	0.5	7
**$PM_{2.5}$**	Feb	1.3	14.5	7.5	0.3*	8
	May	-0.01	-0.1	5.3	0.3*	8
	Aug	-2.1	-23.8	5.1	0.3*	6
	Nov	-1.4	-13.6	6.5	0.4	7

* Indicates performance outside of Emery et al. (2017) suggested benchmarks

Table S4. CMAQ performance metrics for hourly simulated 1×1 km^2 (d03) pollutants compared with AQS observations, reported for the whole domain, the urban core (20 × 20 km^2 area centered on the model domain), and outside the core. For NO_2, additional performance metrics are reported separately for roadside and non-roadside monitoring sites. Roadside sites were defined as monitors located within 100 m of a major roadway (AQS 9 & 10); all other monitors were classified as non-roadside.

Pollutant	Domain	Month	MB	NMB	RMSE	r	Number of AQS
O_3	Whole Domain	Feb	-3.4	-13.2	12.5	0.4*	5
		May	-0.1	-0.3	13.6	0.5	8
		Aug	5.6	20.2*	13.5	0.7	8
		Nov	3.5	20.8*	10.6	0.5	8
	Inside Core	Feb	-6.7	-25.1	14	0.4	2
		May	-2.9	-8.6	13.7	0.4	3
		Aug	-0.04	-0.1	11.9	0.7	3
		Nov	-1.1	-5.5	9.8	0.5	3
	Outside Core	Feb	-2.5	-10	12.1	0.4	3
		May	1.3	4.2	13.5	0.5	5
		Aug	8.5	33.6	14.2	0.7	5
		Nov	4.8	29.2	10.7	0.5	5
NO_2	Whole Domain	Feb	-4.5	-29.1	12.7	0.3	7
		May	-1.2	-14.4	8.3	0.3	7
		Aug	-1.4	-14.7	7.7	0.4	7
		Nov	-3.7	-27	9.3	0.5	7
	Inside Core	Feb	-2	-13.6	11.9	0.3	3
		May	1.7	24.1	8.3	0.3	3
		Aug	2.9	40.1	8.3	0.4	3
		Nov	-1.5	-11	9.1	0.5	3
	Outside Core	Feb	-5.6	-35	13.1	0.4	4
		May	-2.4	-26.5	8.2	0.3	4
		Aug	-3.1	-30.7	7.4	0.5	4
		Nov	-4.6	-33.2	9.4	0.5	4
	Road-side	Feb	-11.1	-53.6	16.8	0.4	2
		May	-6.7	-49.7	10.6	0.4	2
		Aug	-6.1	-44.1	9.9	0.4	2
		Nov	-5.1	-34.5	10.0	0.5	2
	Non_road_side	Feb	-2.1	-15.7	10.9	0.4	5
		May	0.9	13.9	7.1	0.3	5
		Aug	0.5	6.9	6.6	0.5	5
		Nov	-3.1	-23.8	9.0	0.4	5
$PM_{2.5}$	Whole Domain	Feb	1.3	14.5	7.5	0.3*	8
		May	-0.01	-0.1	5.3	0.3*	8
		Aug	-2.1	-23.8	5.1	0.3*	6
		Nov	-1.4	-13.6	6.5	0.4	7
	Inside Core	Feb	1.1	12.1	6.8	0.4	3
		May	0.9	15.4	4.8	0.3	3
		Aug	-1	-12.9	4.7	0.3	2
		Nov	-2	-18	7.1	0.4	2
	Outside Core	Feb	1.3	16.1	7.8	0.3	5
		May	-0.5	-7.8	5.5	0.3	5
		Aug	-2.6	-28.2	5.2	0.4	4
		Nov	-1.2	-11.7	6.2	0.5	5

* Indicates performance outside of Emery et al. (2017) suggested benchmarks

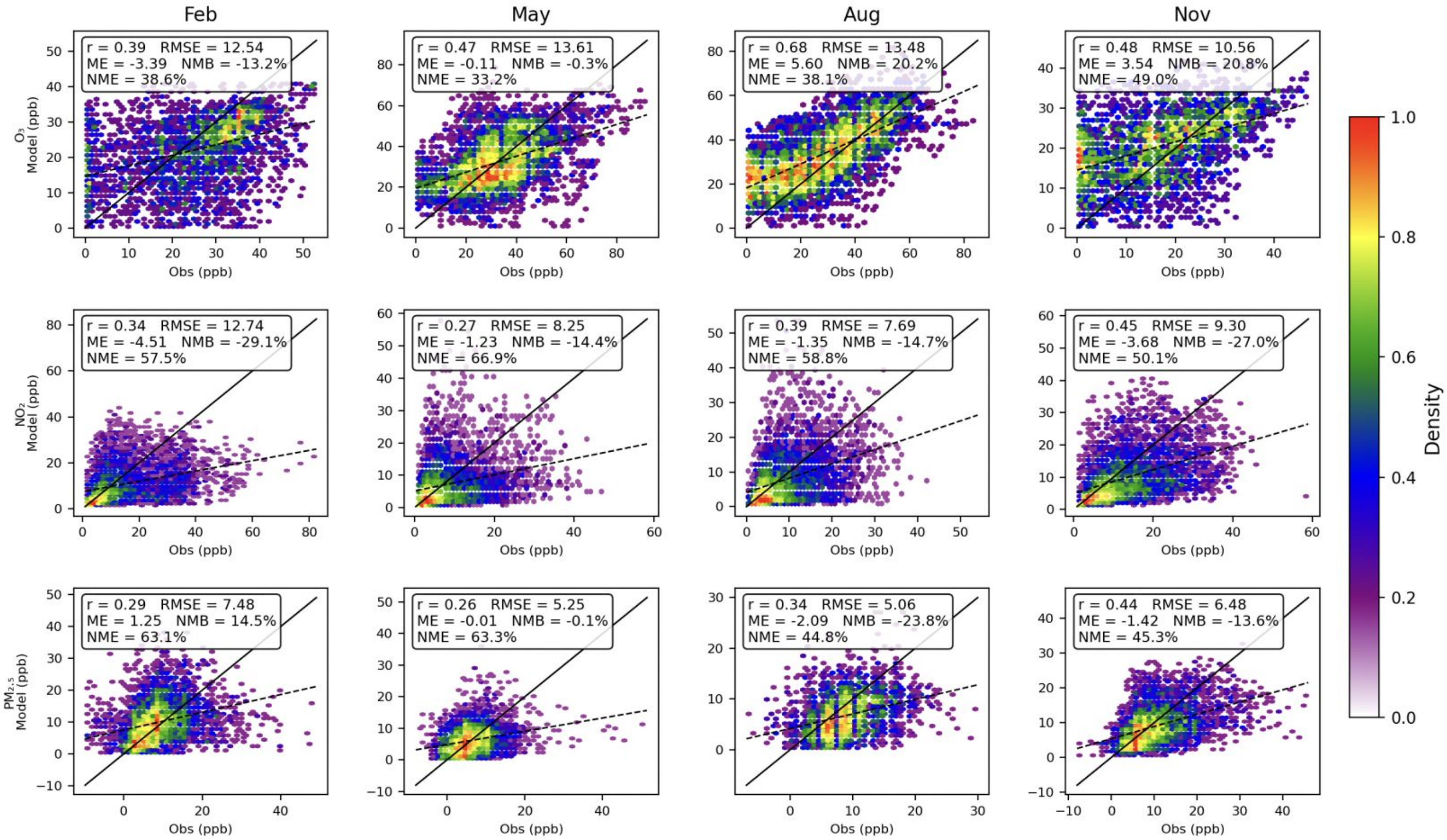

Figure S4: Monthly scatterplot-density comparisons of CMAQ-simulated vs. observed hourly concentrations of O_3, NO_2, and $PM_{2.5}$ for February, May, August, and November.

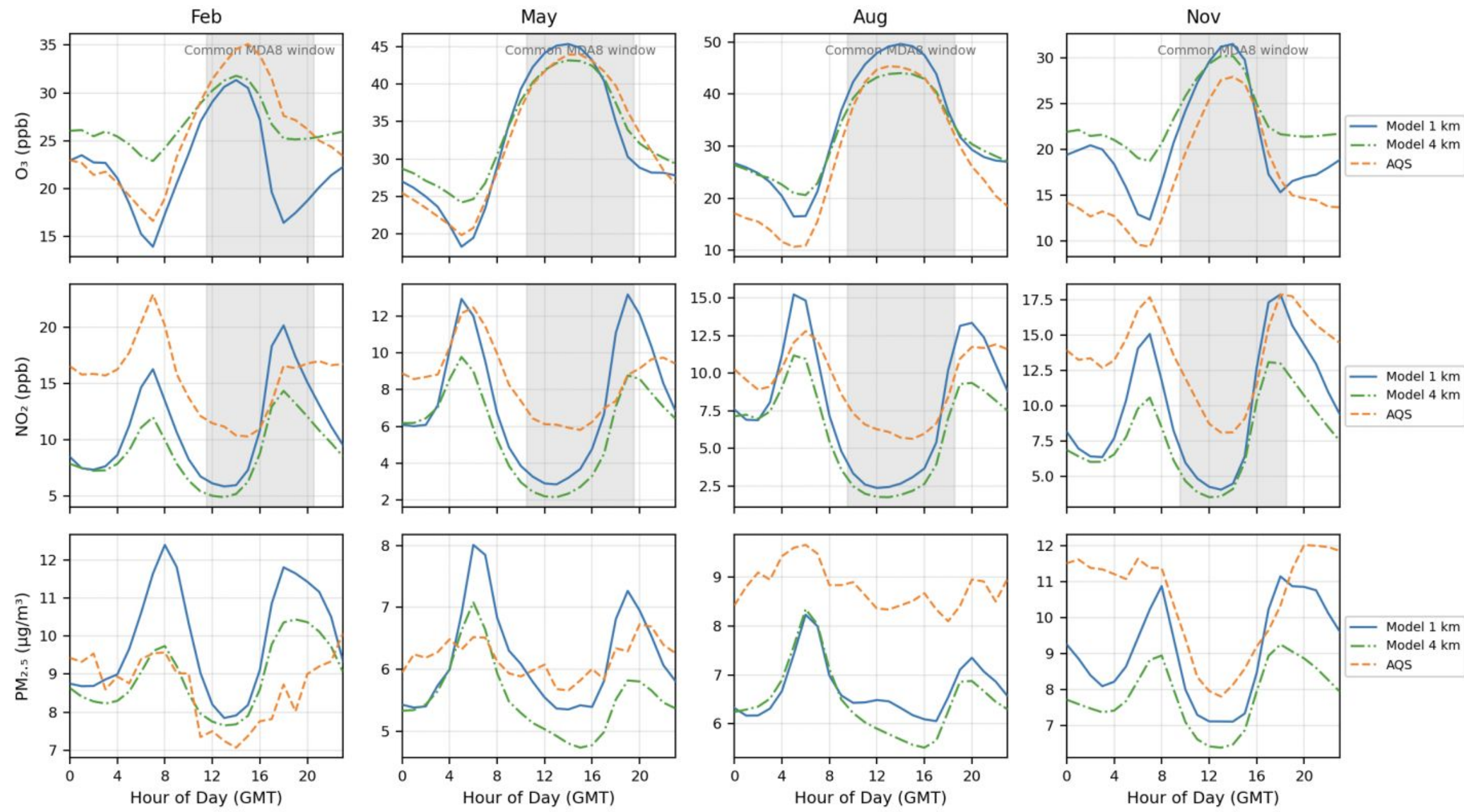

Figure S5. CMAQ (blue for 1×1 km^2 and green dash for 4×4 km^2) and EPA (orange dash) average hourly estimates of pollutants to show the diurnal prediction of pollutants as compared to observations. Gray shading in the O_3 panels denotes the most common hours contributing to the observed MDA8 O_3 metric.

Table S5. NO_2 performance metrics were calculated using the actual observed MDA8 O3-contributing hours for each monitor-day. The hour block shown summarizes the most frequently occurring MDA8 window within each month.

Month	MB	NMB (%)	NME (%)	RSME	r	Most common observed MDA8 O_3-contributing hour block
Feb	1.62	19.66	65.09	8.04	0.44	12-20
May	2.35	54.66	99.84	6.84	0.42	11-19
Aug	0.09	2.16	68.83	4.95	0.37	10-18
Nov	-0.82	-9.42	53.83	6.46	0.41	10-18

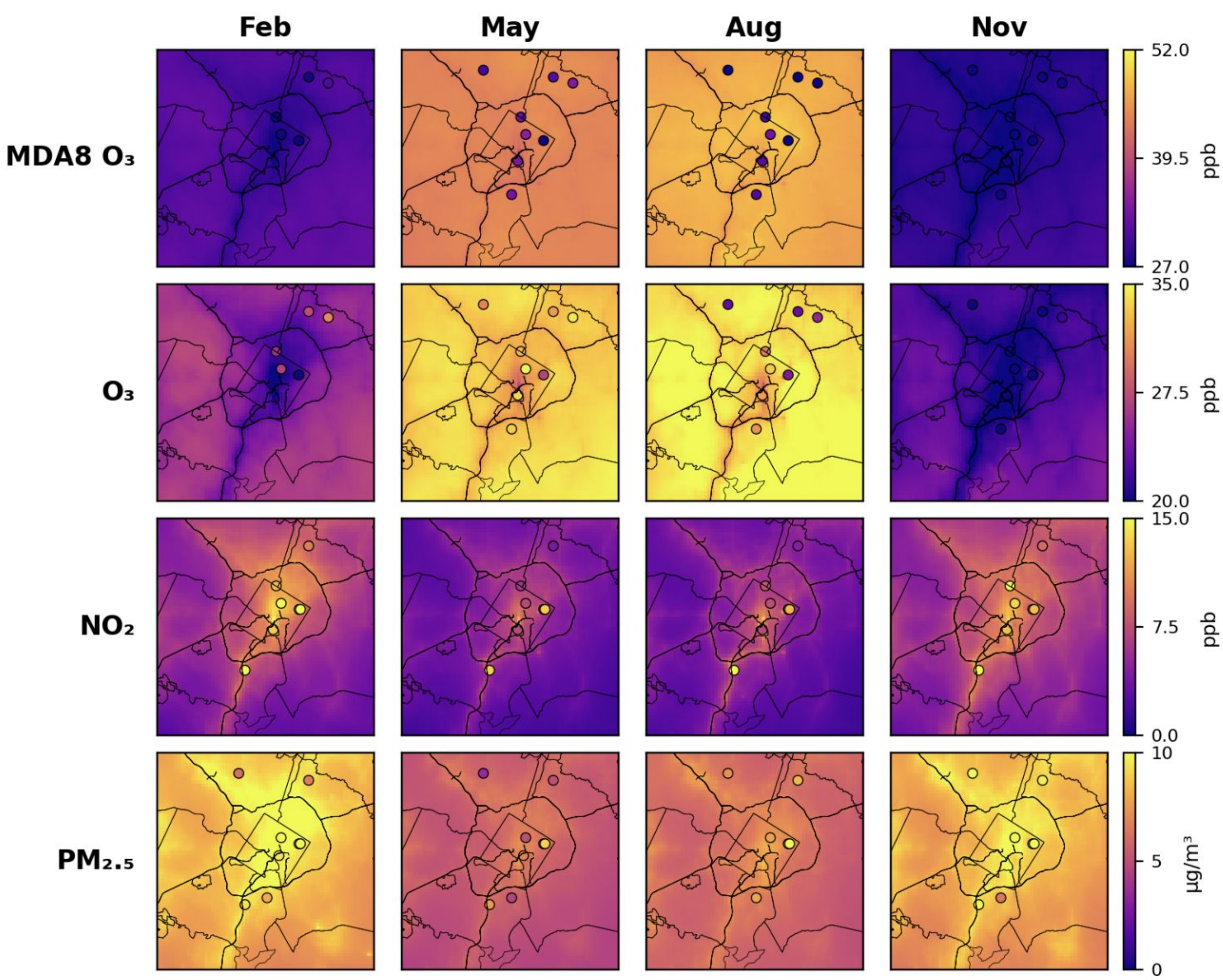

Figure S6. Monthly CMAQ simulation overlaid with station observations.

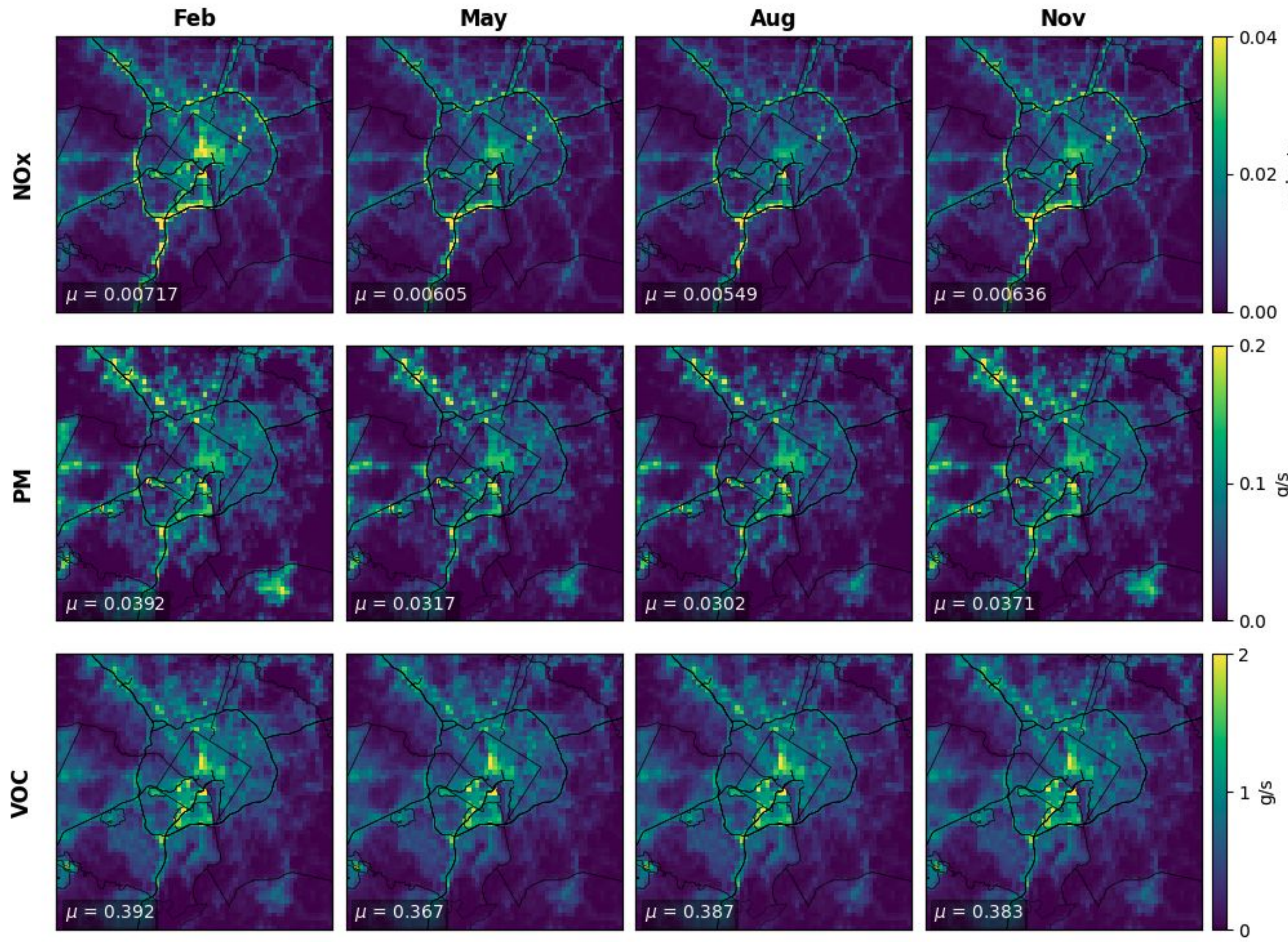

Figure S7. Monthly emissions of NO_x ($NO+NO_2$), PM (EC + OC), and VOCs across the 1×1 km^2 domain (d03)

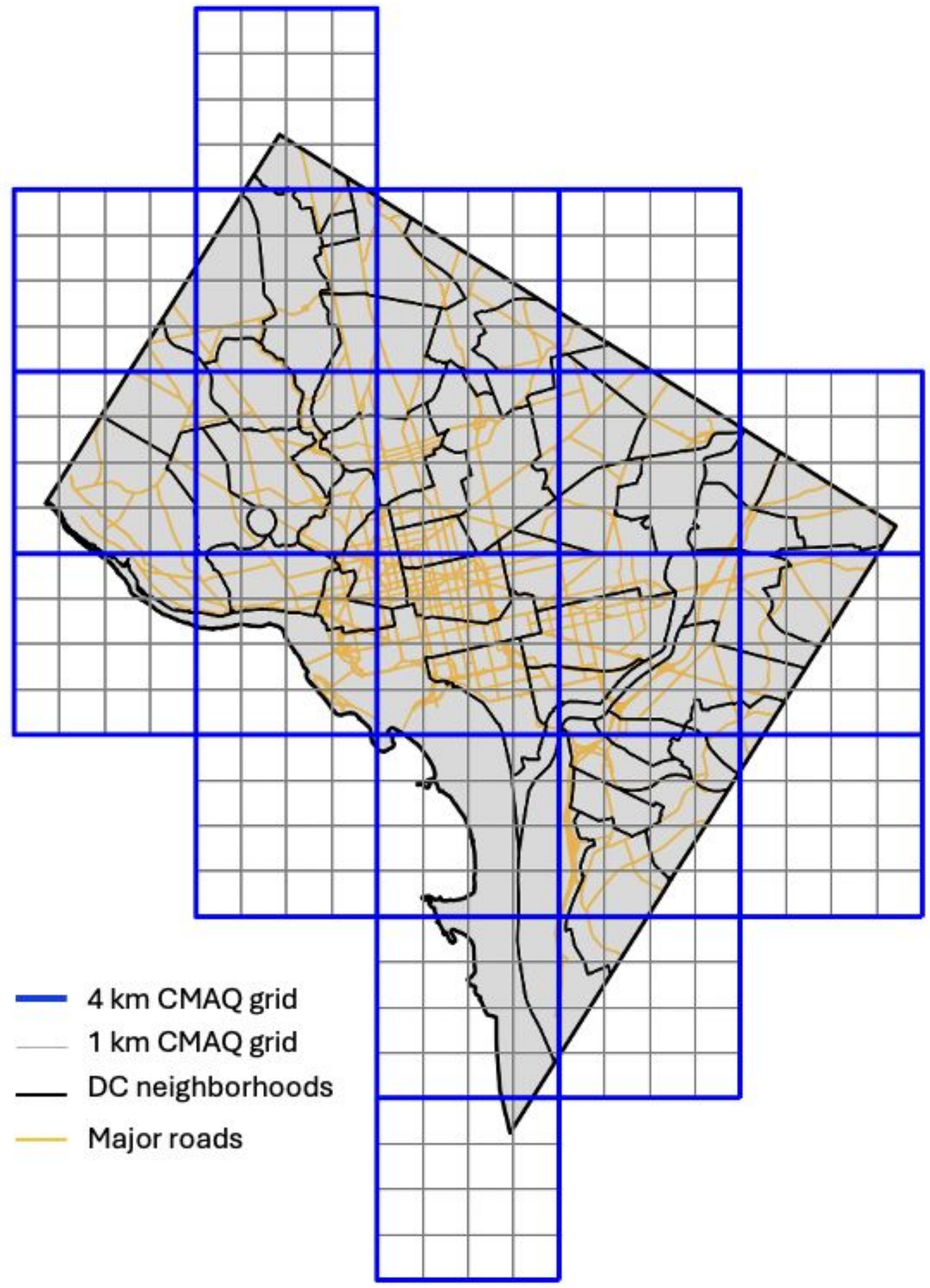

Figure S8. Washington, DC neighborhood boundaries and major roads overlaid with CMAQ model grids at 4×4 km^2 and 1×1 km^2 horizontal resolution. The finer 1×1 km^2 grid is nested within the coarser 4×4 km^2 grid to illustrate spatial refinement over the study domain.

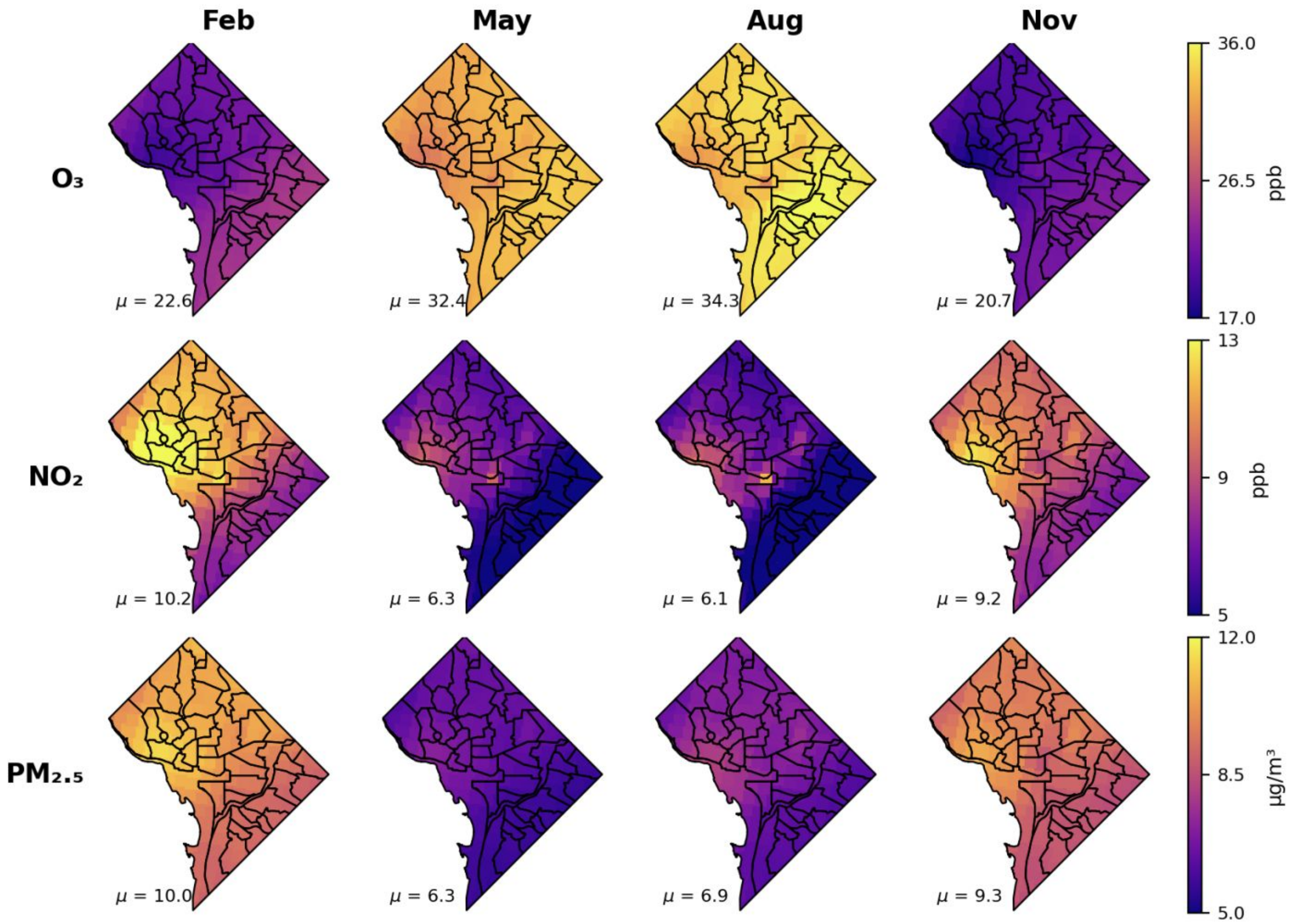

Figure S9. Monthly average O3, NO2, and PM2.5 concentrations over DC, as simulated in the 1×1 km^2 domain. Columns depict Feb, May, Aug and Nov 2017 simulations. DC-average concentrations are annotated (μ)

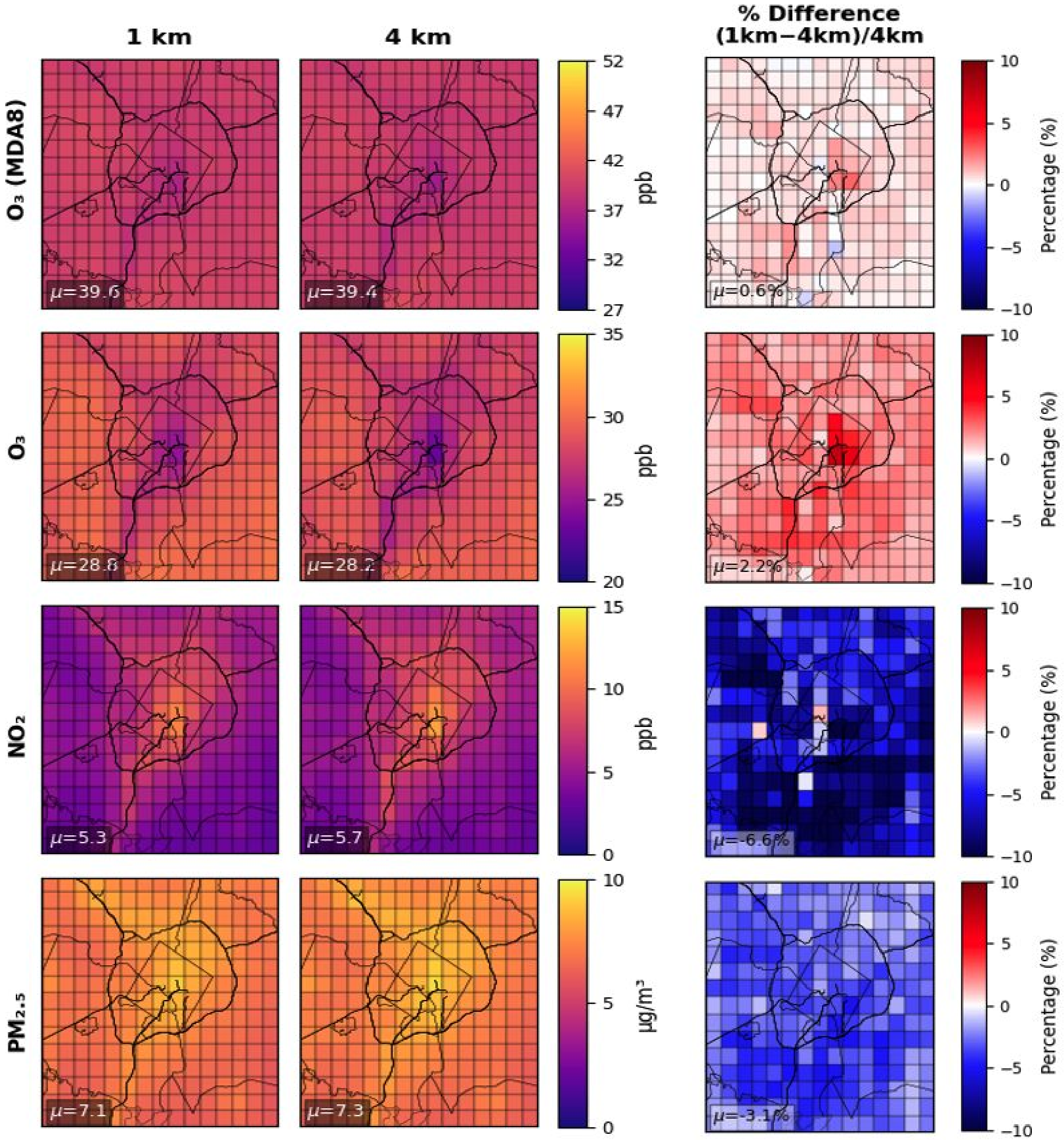

Figure S10. Annual average CMAQ concentrations for MDA8 O_3, daily mean O_3, NO_2, and $PM_{2.5}$ across the full 1x1 km^2 model domain. The first column shows concentrations from the 1x1 km^2 simulation aggregated to the 4x4 km^2 grid, the second column shows concentrations from the native 4x4 km^2 simulation, and the third column shows the percentage difference between the two resolutions.

Table S6. O_3 performance in the 1×1 km^2 domain (d03) assessed against EPA AQS station observations using different O_3 thresholds. The average observed value is noted as μ_d, while the predicted value is noted as μ_p.

O_3 values assessed	Month	μ_d	μ_p	*NMB%*	*NME%*	*r*
**1-hr O_3 > 60 ppb**	**May**	68.3	46.7	-31.6	31.9	0.3
	**Aug**	66.1	52.6	-20.4	21.1	-0.3
**MDA8-hr O_3 > 60 ppb**	**May**	67.3	45.8	-32.0	32.0	0.4
	**Aug**	63.6	48.4	-23.9	23.9	-0.4
**1-hr O_3 > (50%ile Observed O_3)**	**Feb**	35.9	25.8	-28.0	28.7	0.3
	**May**	44.0	36.6	-16.7	26.0	0.4
	**Aug**	41.7	41.1	-1.3	19.2	0.5
	**Nov**	26.4	23.9	-9.4	23.1	0.4
	**Average**	46.3	38.0	-17.5	24.5	0.2
**MDA8-hr O_3**	**Feb**	34.4	28.9	-16.0	22.9	0.2
	**May**	44.3	43.9	-0.8	23.5	0.1
	**Aug**	43.7	46.7	6.9	16.9	0.5
	**Nov**	25.9	27.4	5.8	24.5	0.2
	**Average**	37.1	36.7	-1.0	22.0	0.2

Table S7. Performance of 4×4 km^2 simulation over the 1×1 km^2 (d03) domain, compared to the 1×1 km^2 simulation performance using EPA AQS station data as the observational standard.

	**4×4 km^2**	**Δ (1×1 km^2 - 4×4 km^2)**

Pollutant	Month	ME	NMB	RMSE	r	ΔME	ΔNMB	ΔRMSE	Δr
O_3	Feb	-3.51	-11.6	11.56	0.5	0.12	-1.6	0.98	-0.08
	May	-0.25	-0.7	12.27	0.5	0.14	0.4	1.34	-0.01
	Aug	4.13	14.6	12.57	0.7	1.47	5.6	0.91	0.03
	Nov	1.13	5.1	9.35	0.6	2.41	15.7	1.21	-0.08
NO_2	Feb	-3.42	-27.9	10.26	0.5	-1.09	-1.2	2.49	-0.19
	May	-1.26	-17.9	7.52	0.4	0.03	3.5	0.73	-0.14
	Aug	-1.33	-17.9	7.07	0.5	-0.02	3.2	0.62	-0.09
	Nov	-3.31	-29.9	8.62	0.6	-0.37	2.9	0.68	-0.11
$PM_{2.5}$	Feb	-0.08	-0.9	7.9	0.4	1.33	15.4	-0.5	-0.06
	May	-0.25	-4.2	5.9	0.2*	0.24	4.1	-0.7	0.06
	Aug	-2.27	-25.9	6.3	0.3*	0.18	2.1	-1.26	0.07
	Nov	-0.31	-3.8	6.8	0.4	-1.11	-9.8	-0.37	0.09

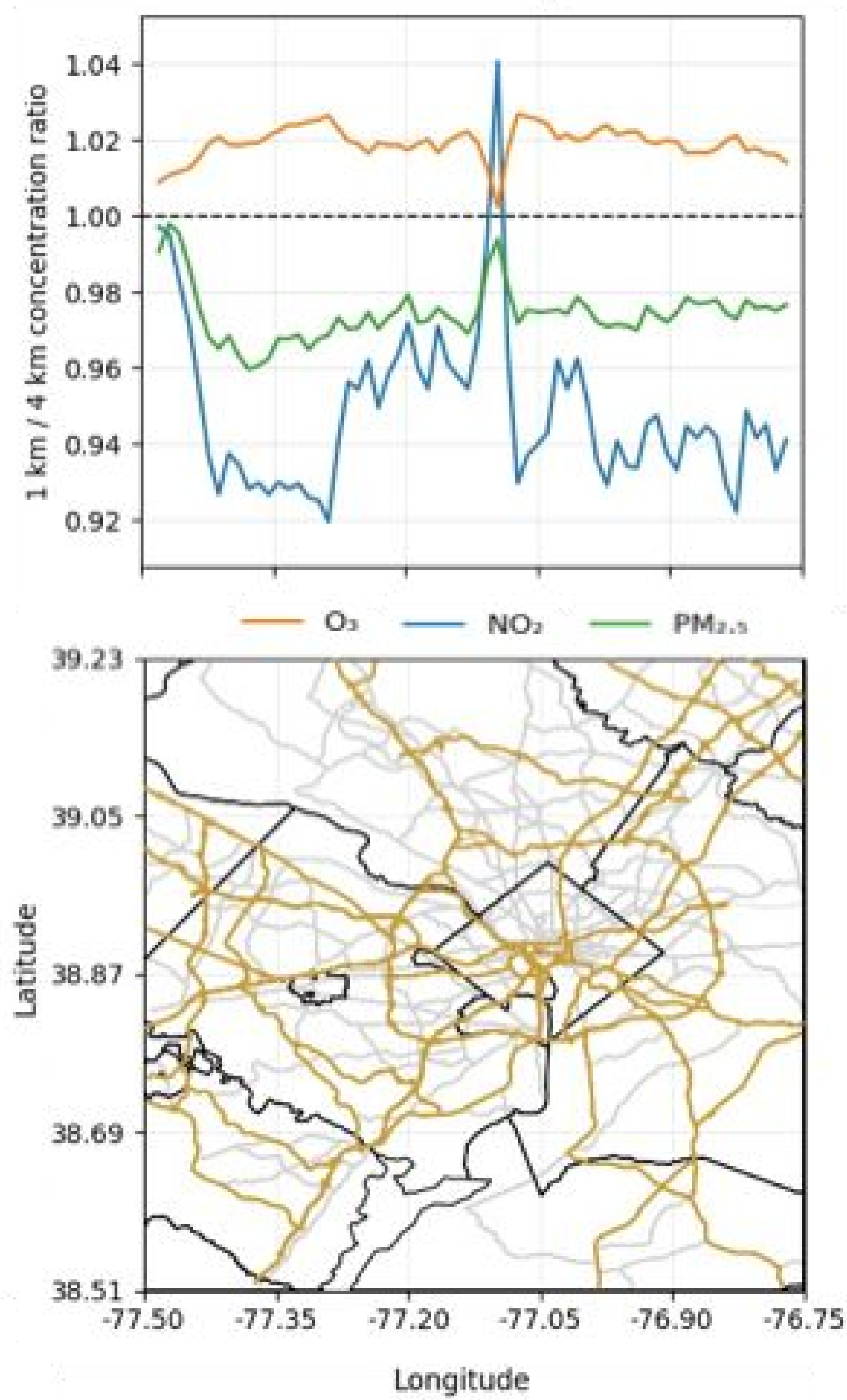

Figure S11. Annual zonal average of O_3, NO_2 and $PM_{2.5}$ across the finest model domain (d03) for both 1×1 km^2 and 4×4 km^2 grid resolutions

Table S8. Studies for the CMAQ comparison and reported metrics of performance.

Study	Domain	Period	Resolution	Pollutant	MB	NMB (%)	NME (%)	r
This study	Washington, DC	4 months (2017)	1 km	O_3	-3 to 6	-13 to 21	33 to 49	0.4 to 0.7
				NO_2	-4.5 to -1.2	-29 to -14	50 to 68	0.3 to 0.5
				$PM_{2.5}$	-2 to 1.3	-24 to 14	45 to 63	0.3 to 0.4
Montgomery et al.,	Chicago region	4 months (2018)	1.3 km	O_3		28 to 58	34 to 63	0.6 to 0.7
				NO_2		-27 to 3	45 to 66	0.5 to 0.6
				$PM_{2.5}$		-38 to 16	51 to 67	0.3 to 0.5
Pan et al., 2017	Houston, TX	2-weeks (2017)	1–4 km	NO_x	-0.6 to 0.1	-	-	0.6 to 0.7
				O_3	10 to 11.4	-	–	0.8 to 0.9

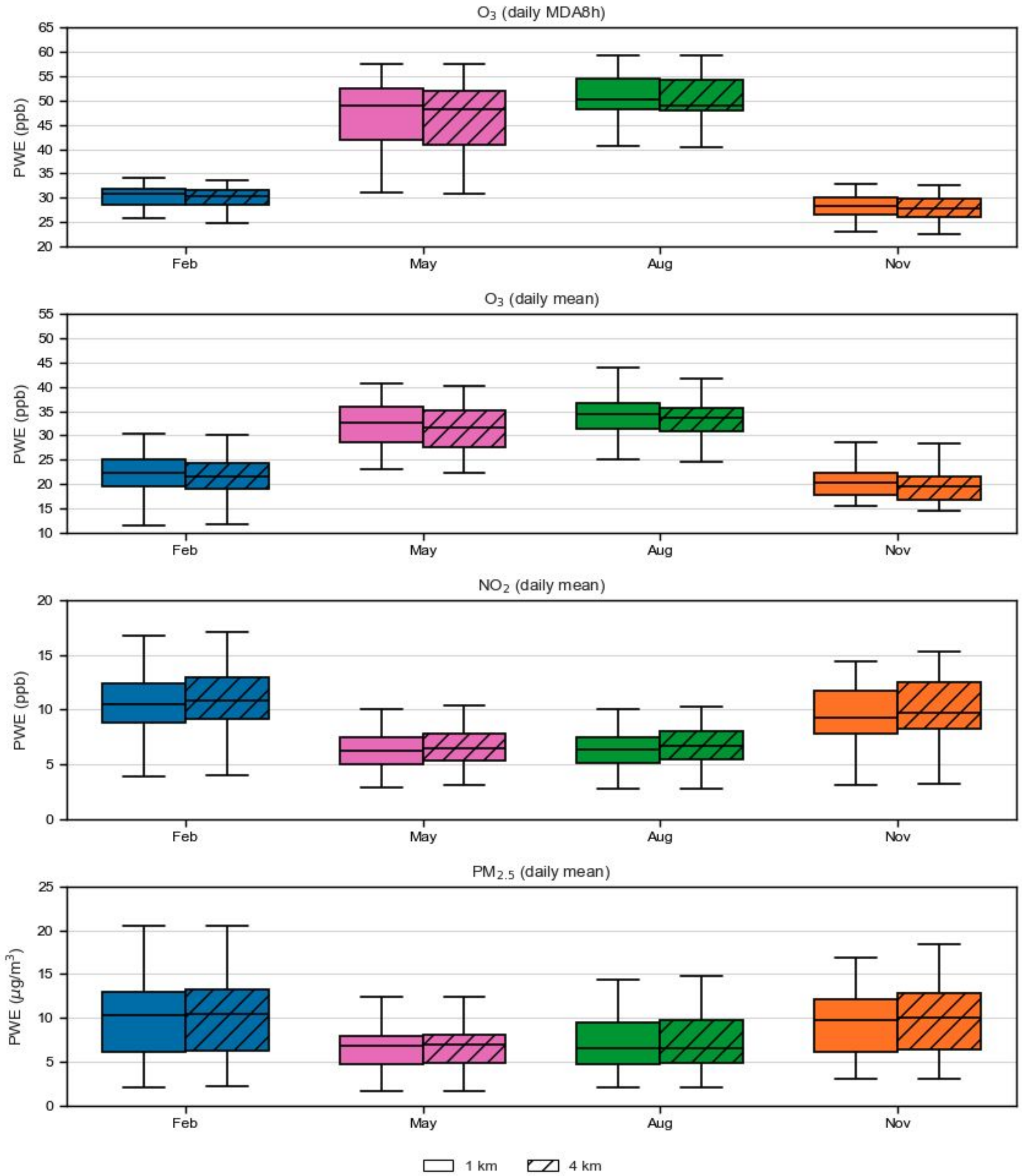

Figure S12. Neighborhood-cluster population-weighted exposure (PWE) across Washington, D.C. Boxplots represent the distribution of daily PWE values for each study month.

Table S9. Reactive VOC species included in the surface VOC/NOx diagnostic. Individual species are represented in CMAQ as mixing ratios (ppbV), converted to carbon mixing ratios (ppbC) using their molecular carbon numbers, and summed to calculate the VOC concentration used in the VOC/NOx analysis. Secondary oxidation products and NOx reservoir species (e.g., NTR and PANs) were excluded because they are formed after VOC oxidation and do not directly represent primary VOC availability.

Seinfeld & Pandis VOC Category	CMAQ Species	Description
Alkanes	ETHA	Ethane
Alkenes	ETH	Ethylene
Aromatics	BENZENE	Benzene
Aromatics	TOL	Toluene
Aromatics	XYL	Xylenes
Aldehydes	FORM	Formaldehyde
Aldehydes	ALD2	Lumped low-molecular-weight aldehydes
Biogenic VOCs	ISOP	Isoprene
Biogenic VOCs	TERP	Monoterpenes

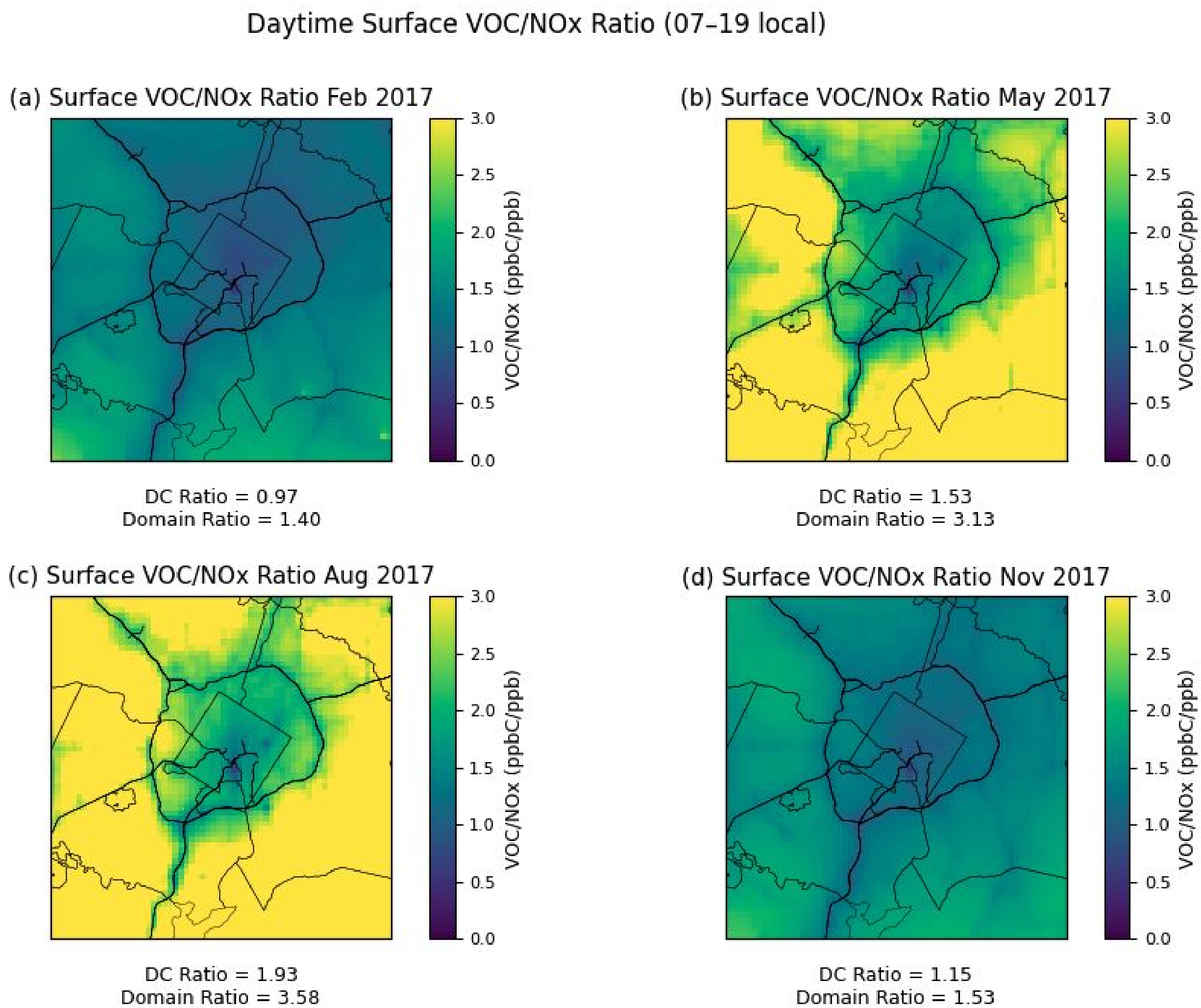

Figure S13. Daytime (7 a.m.–7 p.m.) VOC/NO_x surface ratios for each month of simulation.

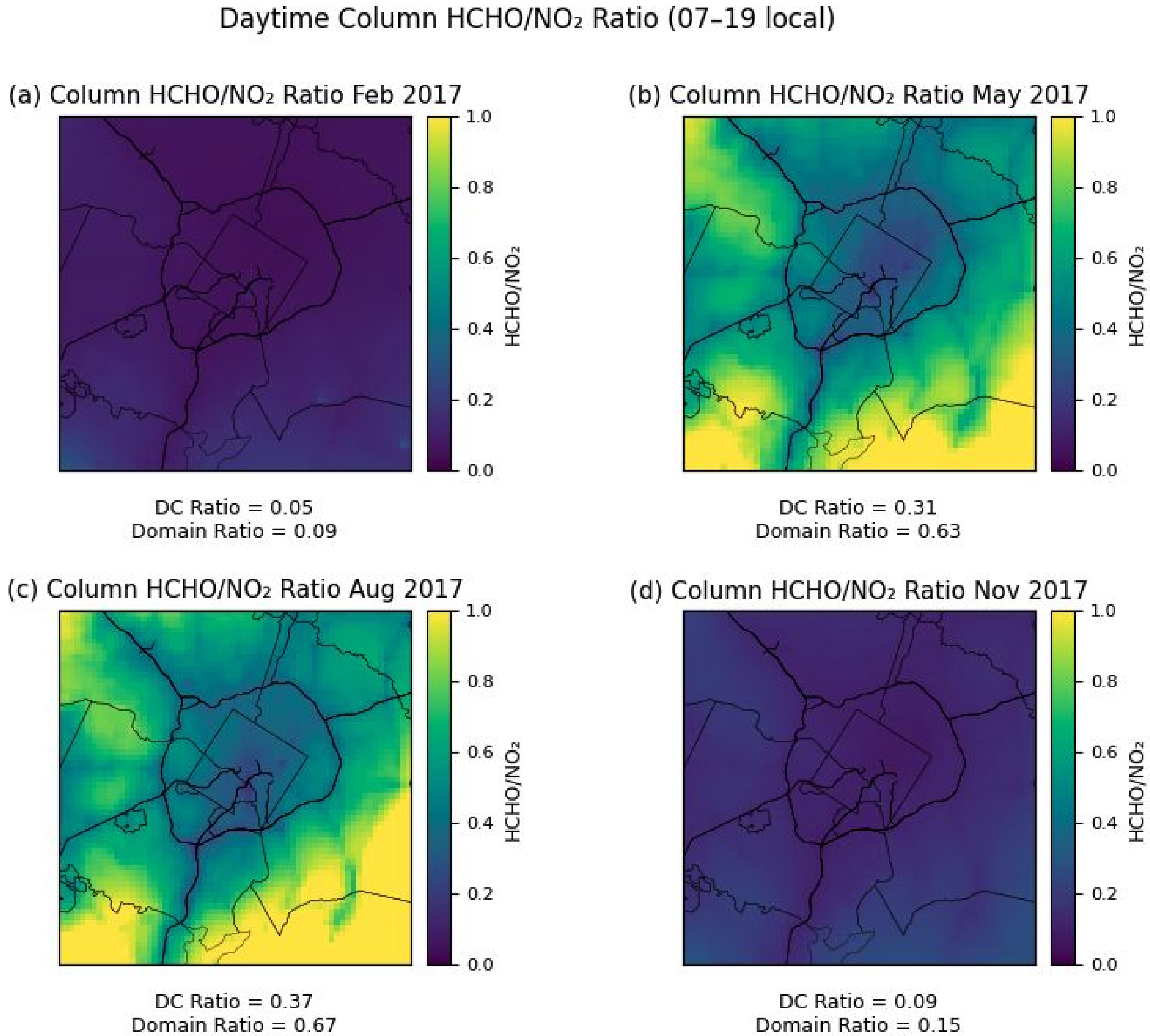

Figure S14. Daytime (7 a.m.–7 p.m.) $HCHO/NO_2$ column ratios for each month of simulation.

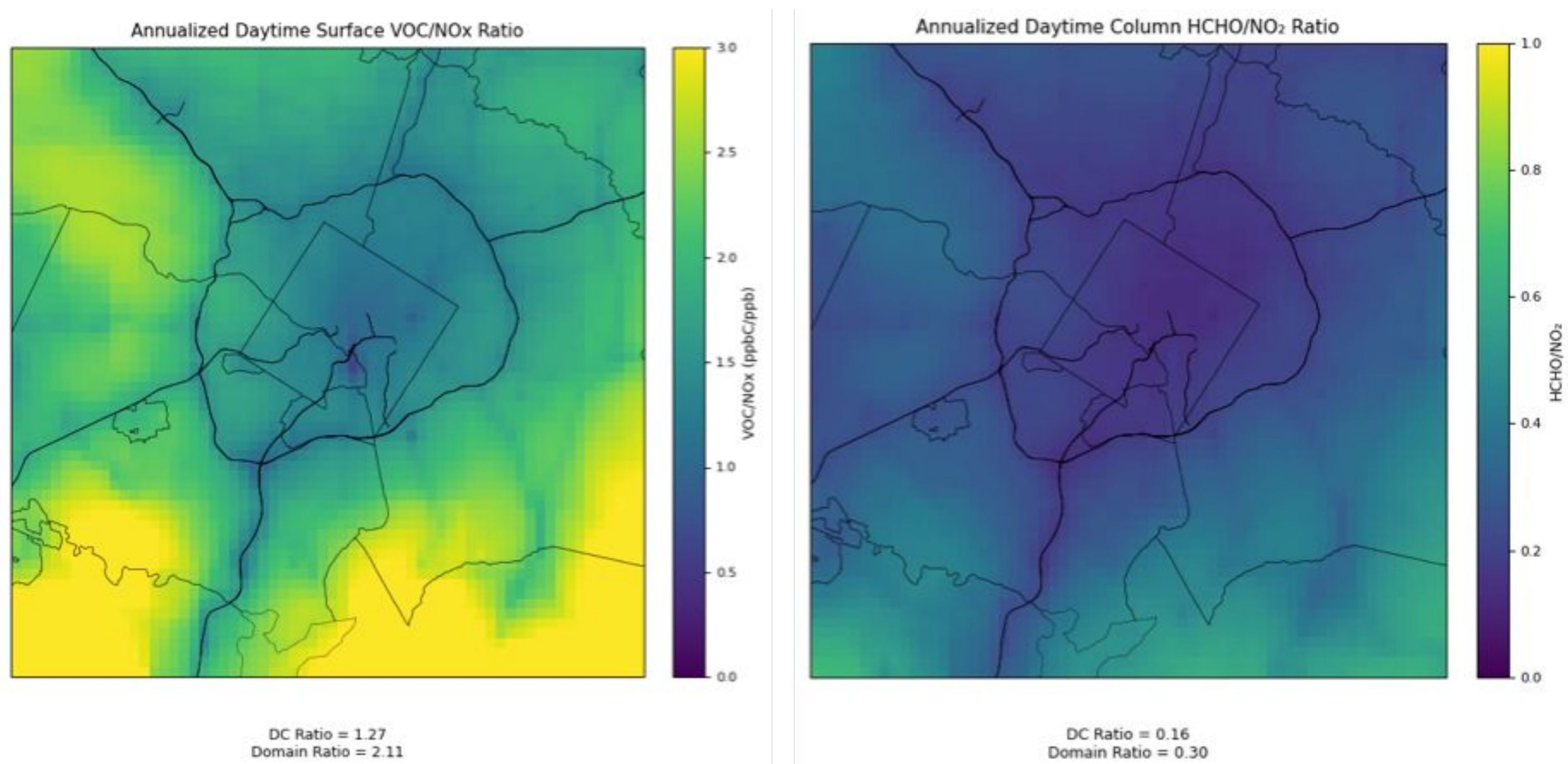

Figure S15. Daytime (7 a.m.–7 p.m.) annualized average of the (a) VOC/NO_x surface ratio and (b) HCHO/NO_2 column ratios over the 1×1 km^2 domain.

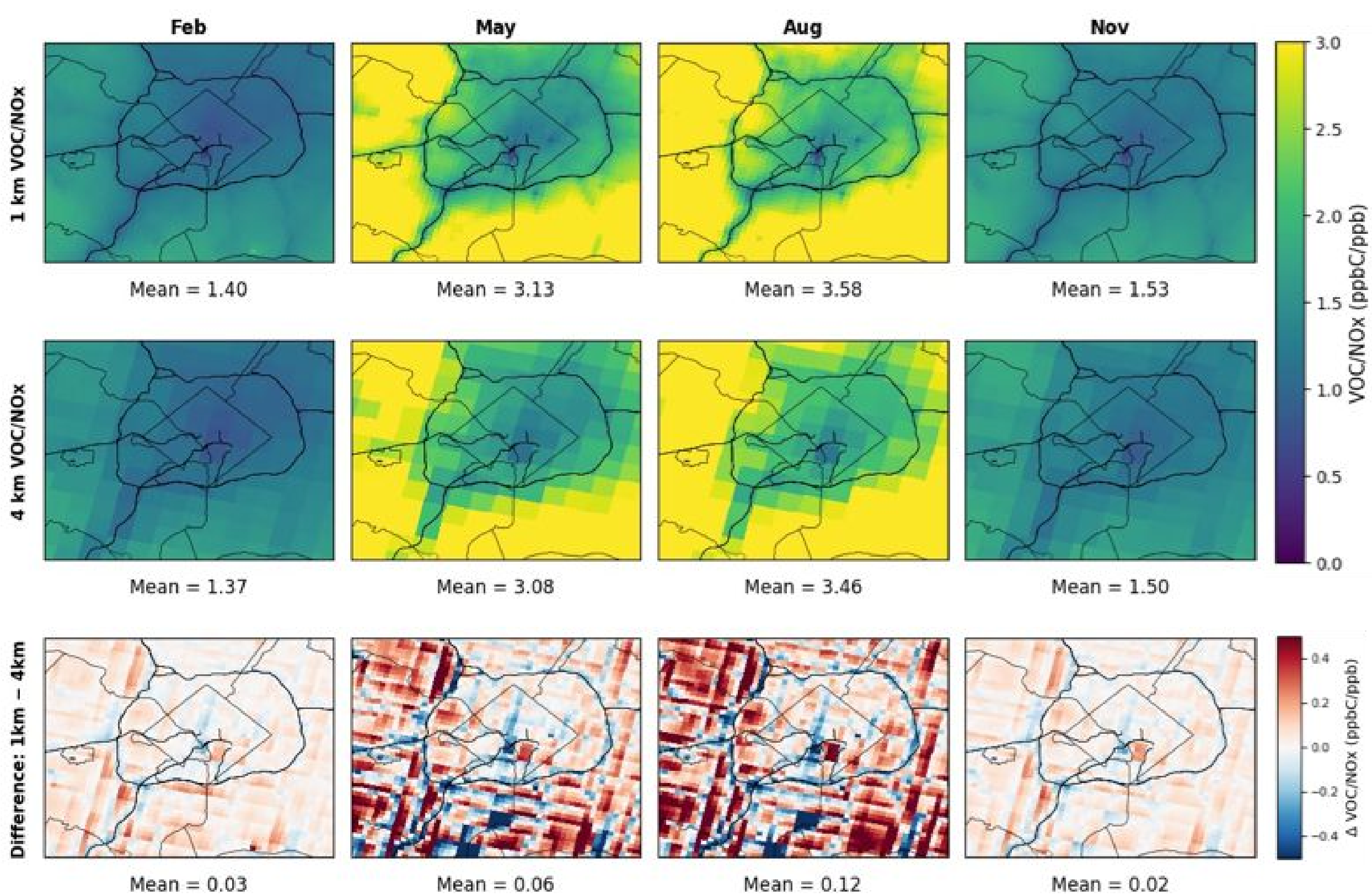

Figure S16. Comparison of Daytime (7 a.m.–7 p.m.) VOC/NO_x Ratios from the 1×1 km^2 and 4×4 km^2 Simulations
